# New information of dopaminergic agents based on quantum chemistry calculations

**DOI:** 10.1038/s41598-020-78446-4

**Published:** 2020-12-09

**Authors:** Guillermo Goode-Romero, Ulrika Winnberg, Laura Domínguez, Ilich A. Ibarra, Rubicelia Vargas, Elisabeth Winnberg, Ana Martínez

**Affiliations:** 1 Departamento de Fisicoquímica, Facultad de Química, Universidad Nacional Autónoma de México, Circuito Exterior SN, Ciudad Universitaria, CP 04510 Ciudad de México, CDMX Mexico; 2 Departamento Académico de Ingeniería Industrial y Operaciones, Instituto Tecnológico Autónomo de México, Río, Hondo 1, Altavista, Álvaro Obregón, CP 01080 Ciudad de México, CDMX Mexico; 3 Laboratorio de Fisicoquímica y Reactividad de Superficies (LaFReS), Instituto de Investigaciones en Materiales, Universidad Nacional Autónoma de México, Circuito Exterior SN, Ciudad Universitaria, CP 04510 Ciudad de México, CDMX Mexico; 4 Departamento de Química, División de Ciencias Básicas e Ingeniería, Universidad Autónoma Metropolitana-Iztapalapa, San Rafael Atlixco 186, Col. Vicentina, Iztapalapa, AP Postal 55-534, CP 09340 Ciudad de México, CDMX Mexico; 5 Department of Health Care Sciences, Ersta Sköndal Bräcke University College, Stigbergsgatan 30, 116 28 Stockholm, Sweden; 6 Departamento de Materiales de Baja Dimensionalidad, Instituto de Investigaciones en Materiales, Universidad Nacional Autónoma de México, Circuito Exterior SN, Ciudad Universitaria, CP 04510 Ciudad de México, CDMX Mexico

**Keywords:** Biophysical chemistry, Biochemistry, Biophysics, Neuroscience, Medical research, Chemistry, Physics

## Abstract

Dopamine is an important neurotransmitter that plays a key role in a wide range of both locomotive and cognitive functions in humans. Disturbances on the dopaminergic system cause, among others, psychosis, Parkinson’s disease and Huntington’s disease. Antipsychotics are drugs that interact primarily with the dopamine receptors and are thus important for the control of psychosis and related disorders. These drugs function as agonists or antagonists and are classified as such in the literature. However, there is still much to learn about the underlying mechanism of action of these drugs. The goal of this investigation is to analyze the intrinsic chemical reactivity, more specifically, the electron donor–acceptor capacity of 217 molecules used as dopaminergic substances, particularly focusing on drugs used to treat psychosis. We analyzed 86 molecules categorized as agonists and 131 molecules classified as antagonists, applying Density Functional Theory calculations. Results show that most of the agonists are electron donors, as is dopamine, whereas most of the antagonists are electron acceptors. Therefore, a new characterization based on the electron transfer capacity is proposed in this study. This new classification can guide the clinical decision-making process based on the physiopathological knowledge of the dopaminergic diseases.

## Introduction

During the second half of the last century, a movement referred to as the third revolution in psychiatry emerged, directly related to the development of new antipsychotic drugs for the treatment of psychosis. Treatment of psychosis has evolved with the development of antipsychotic drugs. The dopamine hypothesis, which defines the physiological mechanism of schizophrenia (a type of psychosis) postulates that this is derived from a primary imbalance in the dopaminergic system^1–44^. Currently, there are at least eleven different types of dopaminergic drugs for the control of psychotic symptoms. To date, all drugs with antipsychotic efficacy show some affinity and activity at the D2 subtype of the dopamine receptor^36^.

Research focusing on new antipsychotics has led to greater knowledge on their biochemical effects; however, the physiological mechanism of action underlying their pharmacological therapy still requires explanation. For the most part, antipsychotics can be classified as antagonists or agonists, according to their functionality. Antagonist drugs are those that bind to receptors, in this case dopamine receptors and block them, while agonist drugs are those that interact with the receptors, thereby activating them. An agonist produces a conformational change in the dopamine receptors (coupled to a G-protein) that turns on the synthesis of a second messenger. Antagonists also produce a conformational change in the receptor but without change in signal transduction.

Experimentally, drugs are classified as either agonists or antagonists based on complex behavioral analysis, as well as rotational experiments with rats^25,38,39^. In addition to agonist–antagonist classification, antipsychotics have been classified according to having affinity for more than one receptor subtype, leading to first and second-generation of antipsychotics^40^.

Previous reports^45–47^ have used quantum chemistry calculations to help describe the pharmacodynamics of antipsychotic drugs, relating biological activity to chemical reactivity indices, such as chemical hardness and first ionization energy. There is also a comparative study of 32 oral antipsychotics used for treatment of schizophrenia (3 partial agonists and 29 antagonists) recently published^48^. Authors report specific aspects for the antipsychotics such as efficacy, quality of life and side effects. They conclude that, because so many antipsychotics options are available, this analysis should help to find the most suitable drug for each patient. They also found efficacy differences between molecules, but drugs differ more in their side effects than in the effectiveness. It is clear that more research is needed to explain the psychopharmacodynamic effect these drugs have.

In spite of all existing research on dopaminergic agents, to date, very little empirical and theoretical data exist to elucidate mechanisms of action. Based on the idea that all molecules have chemical properties that can be described in terms of response functions related to chemical reactivity, the principal aim of this investigation is to examine 86 molecules classified as agonists and 131 molecules classified as antagonists (Tables 1, 2) by applying Density Functional Theory (DFT) calculations. We analyzed electron transfer capacity as a response function, because it can be related to the pharmacodynamics of the molecules that control electrochemical signaling in cells, a function which is imbalanced during e.g. psychosis, Parkinson’s disease and Huntington’s disease. The aim of the study is to explore the intrinsic properties of D2 ligands without the receptor, in an effort to predict some of their inherent characteristics prior to any biological interactions. We hypothesize that the dichotomy behavior of electron donation or acceptance provides an interesting and more precise way to classify ligands than the conventional agonist/antagonist biological profile.

**Table 1:** Conventional classification of dopaminergic agents that are agonists reported in alphabetical order.

5OH-DPAT	Bifeprunox	Dihydroergocryptine	Lisuride	Quinpirole
6Br-APB	(*R*)-Boldine	Dihydroergotamine	Mesulergine	RDS127
7OH-DPAT	(*S*)-Boldine	Dinapsoline	Methylphenidate	RO105824
7OH-PIPAT	Blonanserin	Ergocornine	Minaprine	Ropinirole
8OH-DPAT	Brexpiprazole	α-Ergocryptine	(*R*)-Nuciferine	Rotigotine
A412997	Brasofensine	β-Ergocryptine	OSU6162	SKF38393
A77636	Brilaroxazine	α-Ergosine	PD128907	SKF77434
A86929	Bromocryptine	β-Ergosine	PD168077	SKF81297
ACP104	(*R*)-Bulbocapnine	Ergometrine	Pergolide	SKF82958
Alentemol	(*S*)-Bulbocapnine	Ergotamine	PF216061	SKF83959
(*S*)-Amphetamine	Cabergoline	Epicryptine	PF592379	SKF89145
Aplindore	Cariprazine	Fenoldopam	Pardoprunox	Stepholidine
(*R*)-Apomorphine	Chanoclavine I	Flibanserin	Piribedil	Sumanirole
(*S*)-Apomorphine	*cis*8-OH-PBZI	(*R*)-Glaucine	Pramipexole	Talipexole
(*R*)-Aporphine	Dihydrexidine	(*S*)-Glaucine	(*R*)-Pukateine	Trepipam
(*S*)-Aporphine	Dihydroergocornine	Hordenine	Quinagolide	Vilazodone
Aripiprazole	Dihydroergocristine	Lergotrile	Quinelorane	Zelandopam
Bicifadine

**Table 2:** Conventional classification of dopaminergic agents that are antagonists, reported in alphabetical order.

Abaperidone	Cisapride	Imipramine	Olanzapine	Sertindole
Aceperone	Clebopride	Itopride	Paliperidone	Setoperone
Acepromazine	Cloroperone	Lenperone	Pentiapine	S142907
Acetophenazine	Clotiapine	Levomepromazine	Perphenazine	SCH23390
Alizapride	Clozapine	Lodiperone	Perospirone	Spiperone
Amiperone	Cyclindole	Loxapine	Pimavanserin	Spiroxatrine
Amisulpride	Declenperone	Lumateperone	Pimethixene	Sulpiride
Amoxapine	Desipramine	Lurasidone	Pimozide	Tefluthizol
Aptazapine	Diethazine	Mafoprazine	Pipamperone	Tenilapine
Asenapine	Dixyrazine	Mazapertine	Pipothiazine	Tetrabenazine
Azabuperone	Domperidone	Melperone	Prideperone	Thiethylperazine
Azaperone	Dothiepin	Mequitazine	Primaperone	Thioridazine
Batanopride	Droperidol	Mesoridazine	Proclorperazine	Thiothixene
Benperidol	Ecopipam	Metoclopramide	Promethazine	Tiapride
Biriperone	Enciprazine	Metopimazine	Propiomazine	Timiperone
BL1020	Etoperidone	Metrenperone	Propyperone	Tiospirone
Bromopride	Fananserin	Mindoperone	Quetiapine	Trifluoperazine
Bromperidol	Flucindole	Mirtazapine	Raclopride	Trifluperidol
Buspirone	Fluphenazine	Molindone	Remoxipride	UH232
Carperone	Flumezapine	Moperone	Renzapride	Veralipride
Carphenazine	Flupenthixol	Mosapride	Rilapine	Yohimbine
Chlorpromazine	Fluperlapine	Nafadotride	Risperidone	Zacopride
Chlorprothixene	Gevotroline	Nemonapride	Roxindole	Zetidoline
Cicarperone	Haloperidol	Nonaperone	Roxoperone	Zicronapine
Cinitapride	Homopipramol	Nortriptyline	Sarizotan	Ziprasidone
Cinuperone	Iloperidone	Ocaperidone	Seridopidine	Zoloperone
				Zuclopenthixol

## Results

The hypothesis underlying our investigation is that agonist molecules have electron transfer properties similar to those of dopamine; whereas antagonists of dopamine have a different capacity to transfer charge. At molecular level, this may explain why antagonists bind to the receptors without activating them.

### DAM of all studied compounds

**Figure 1:**
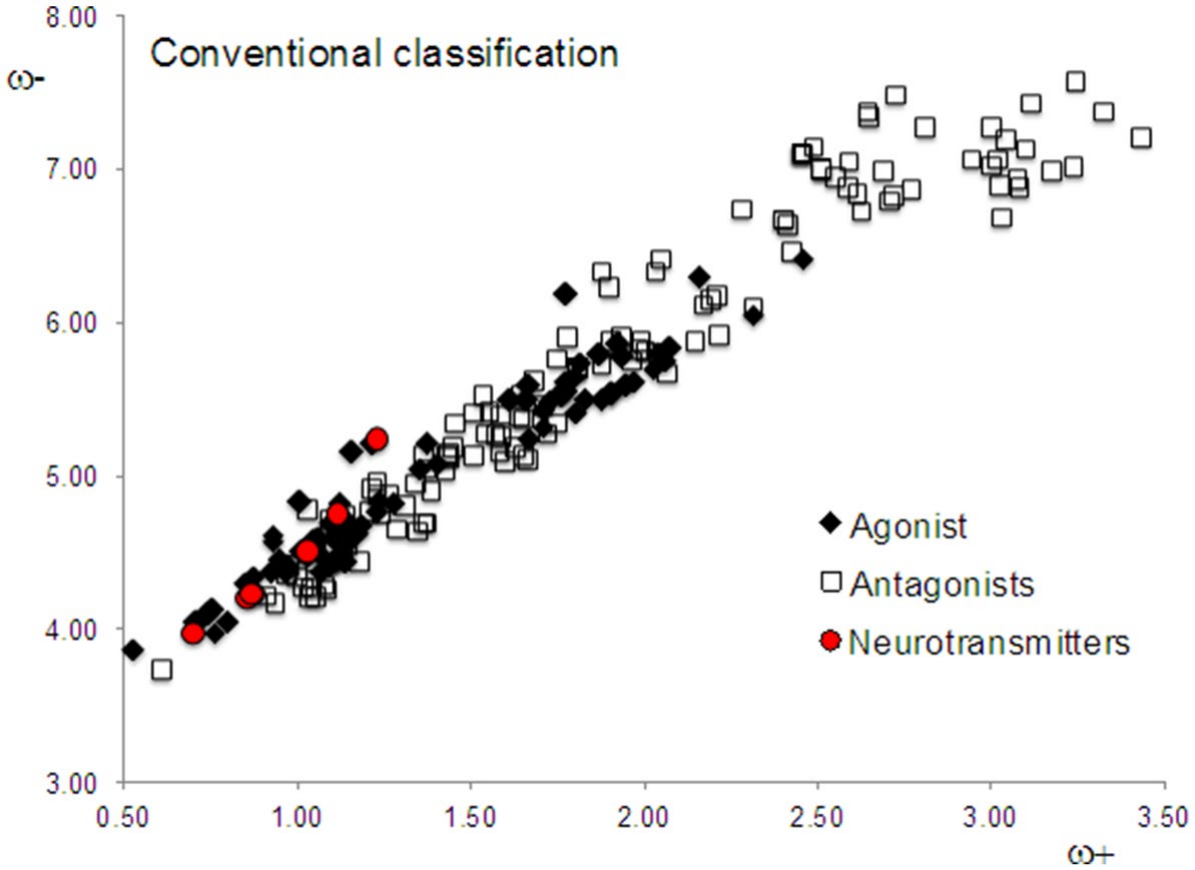
DAM of all the studied compounds. Neurotransmitters are a reference group that includes dopamine and derivatives of dopamine with pharmacological related activity.

We calculated the electrodonating and electroaccepting powers (ω^−^ and ω^+^) of the endogenous neurotransmitter dopamine and the related compounds dopexamine, epinine, etilevodopa, ibopamine, levodopa and melevodopa, as well as dopaminergic ligands and closely related substances (86 agonists and 131 antagonists) in order to analyze their electron transfer properties. Dopamine and related compounds are calculated in order to compare their electron transfer properties with that of the pharmaceuticals studied (Table 3). The results are described in Fig. 1, where we present the DAM of all ligands including the neurotransmitter group. Black squares represent so-called agonists, whereas white squares represent antagonists (see Tables 1, 2). Evidently, there is no clear difference between these two and it is apparent that there are many exceptions to our hypothesis. There are several agonists that are not as good electron donors as dopamine and contrarily, there are many antagonists that have similar electron donor properties to dopamine.

**Table 3:** Data of neurotransmitter dopamine and related compounds are reported.

Name	ω^+^	ω^−^	Notes
Dopamine	0.87	4.23	Endogenous agonist at dopamine receptor subtypes D_1_, D_2_, D_3_, D_4_ and D_5_ receptors
Dopexamine	0.86	4.20	D_2_ full agonist
Epinine	0.87	4.23	Dopaminergic agonist
Etilevodopa	4.50	1.03	Prodrug of dopamine
Ibopamine	5.24	1.23	Prodrug of dopamine
Levodopa	0.70	3.96	Precursor of dopamine
Melevodopa	1.12	4.75	Prodrug of dopamine

### Family I of compounds

**Figure 2:**
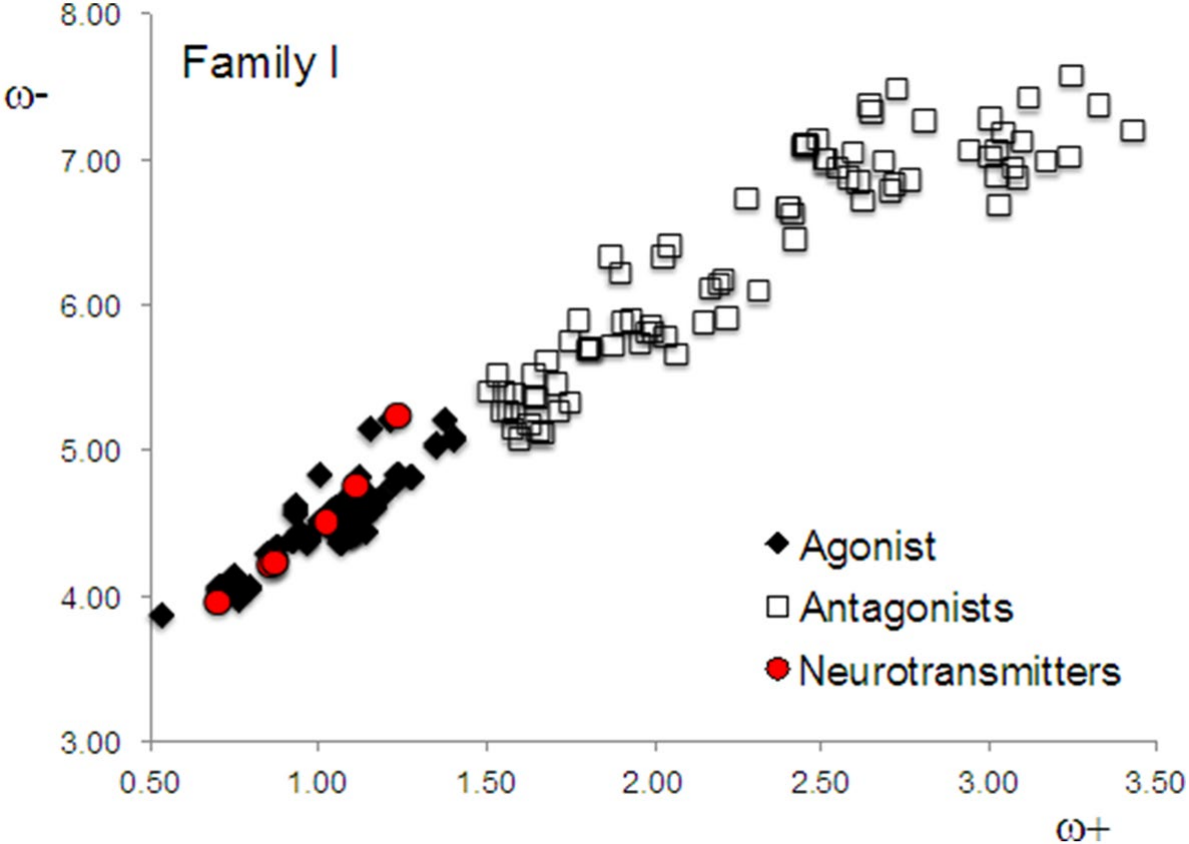
DAM of Family I.

Analyzing the information available concerning the characteristics of these drugs, it turns out that certain molecules are neither exclusively agonists nor exclusively antagonists of D2 dopamine (complete list of references are given in Supplementary Information). They bind to multiple receptors or they are used as antidepressants, or they can act as either agonists and/or antagonists, depending on dosage. In order to analyze these results more carefully, we divided the system into two new families. Family I consists of those dopamine receptor ligands that can be easily characterized as either agonists or antagonists, and mainly bind to the D2 receptor of dopamine. In this family, there are 54 molecules classified as agonists and 88 molecules classified as antagonists. The DAM of Family I is reported in Fig. 2 and evidently the ordering is impressive. Apparently, these agonists have values of ω^+^ that are lower or equal to 1.5 and the antagonists of this family have values of ω^+^ higher than 1.5. All agonists are close to dopamine and the neurotransmitter group, and they are also better electron donors than the antagonists. Antagonists are good electron acceptors in contrast to dopamine, which is a good electron donor. Taking this set of molecules, we can conclude that agonists have similar electron transfer capacity to dopamine, whereas antagonists differ from dopamine in this sense.

### Family II of compounds

Family II comprises 76 molecules that are reported as “partial” or “weak” agonists or antagonists, and some of them present binding affinity for multiple receptors. Regardless of whether they are reported as “weak” or “partial” agonists/antagonists, these molecules were included in the conventional classification of agonists/antagonists with antiparkinsonian or antipsychotic effects. Family II form a group that is heterogeneous, with molecules that have affinity for multiple receptors and they are also weak or partial agonists or antagonists. They do not present selectivity to dopamine receptors*.*

**Figure 3:**
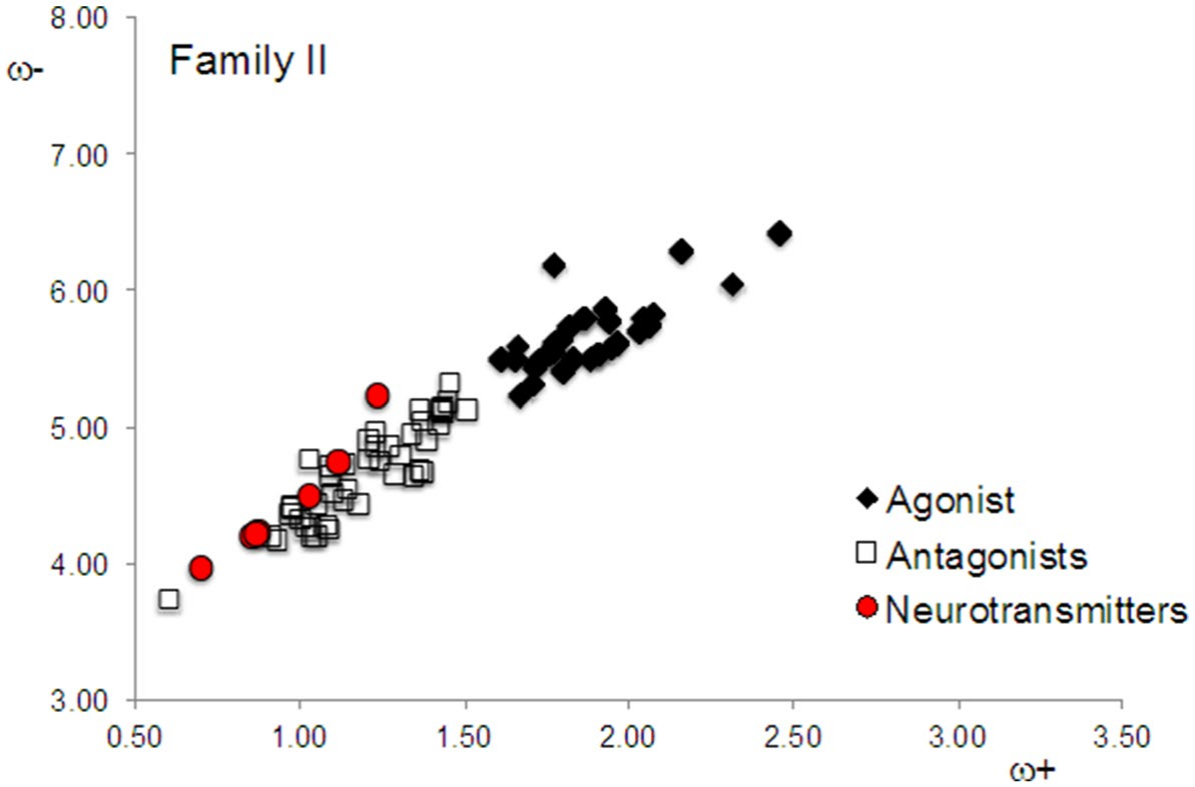
DAM of Family II.

The DAM of Family II is included in Fig. 3. Surprisingly, the tendency is inverted, *i.e.* antagonists have similar electron donor properties to dopamine, whereas agonists have different electron donor properties. It is important to emphasize that previously reported experimental data concerning the reactivity of these molecules is either imprecise or indicates that these molecules bind to multiple receptors. The inverse association found in Family II is difficult to explain, but may be an indication of the complications related to the experimental classification of these drugs. The inherent uncertainty associated with the ex vivo or in vivo experiments is a non-parametric entity that is composed of at least two levels of contributions: the supramolecular and the organellar-cellular. The supramolecular contribution of that uncertainty is related to the lack of abstraction, or “isolation”, of the modeled system being studied (i.e., interference from other proteins that interact with the receptor, presence of some ligands, significant changes to membrane composition, etcetera). The organellar-cellular contribution of this uncertainty is a “background-noise-like" factor, related to variation in the post-translational modifications of proteins, assimilation of the response signals by several cellular components, termination of these signals by natural mechanisms, among others.

## Discussion

**Figure 4:**
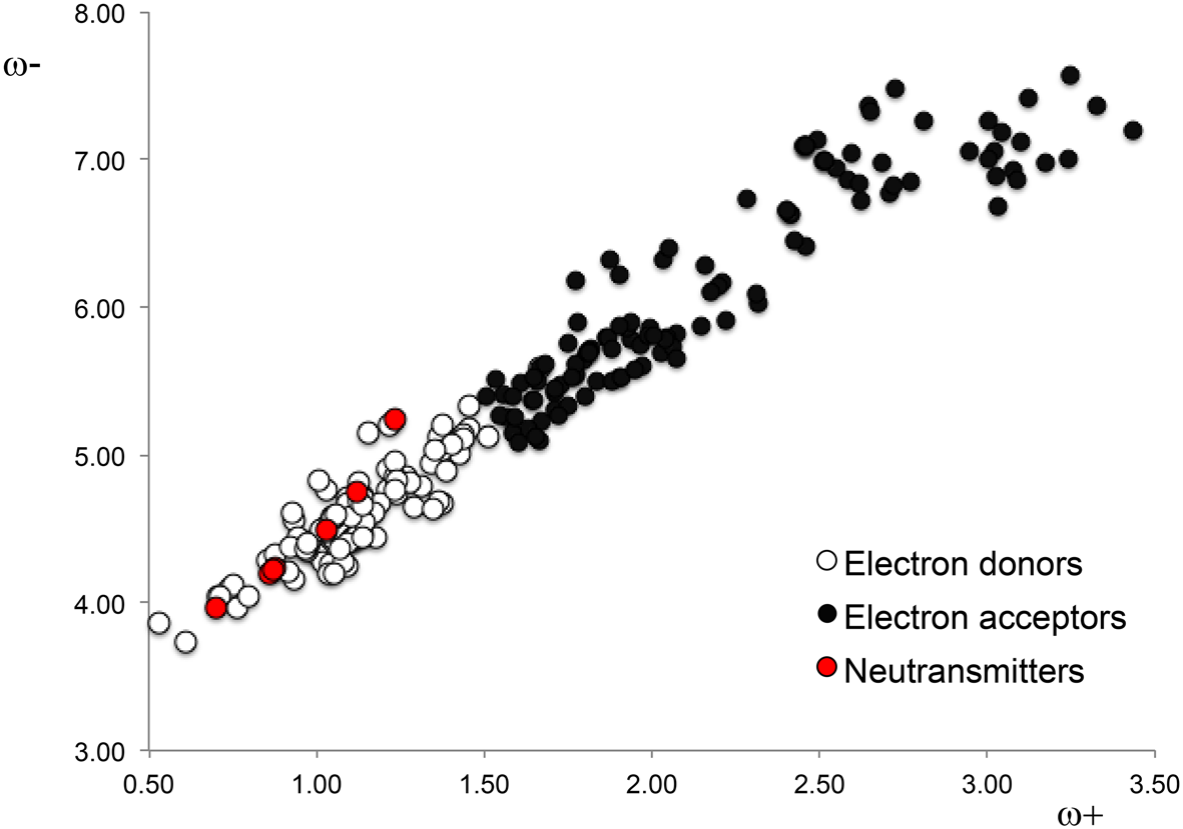
DAM of all compounds considering the information of Tables 4 and 5.

Importantly, behavioral experiments undertaken with rats manifest a degree of ambiguity, inherent to the complexity of biological systems and also to the evaluation and interpretation of data. This degree of ambiguity is not present in quantum chemistry calculations. The hypothesis here is that drugs with electron-transfer properties similar to neurotransmitters will also manifest similar action mechanisms. We thus report new information about the electron donor–acceptor properties of the molecules. This new information is presented in Tables 4 and 5 with specific order. The dopamine receptor ligands with ω^+^ values below or equal to 1.5 are electron donors and those with ω^+^ values greater than 1.5 are electron acceptors. This new information generated the DAM reported in Fig. 4. We also included neurotransmitter-related molecules that constitute good electron donors (Table 3). The value of 1.5 for ω^+^ is arbitrary, but this number emerges when we consider experimental information related to the characterization of agonists and antagonists. Within this range, experimental information concurs with theoretical values because all adequately characterized agonists present ω^+^ values that are less or equal to 1.5, and all adequately characterized antagonists manifest values that exceed a ω^+^ value of 1.5. This enabled us to classify the molecules with reference to reported experimental and theoretical information.

**Table 4:** Pharmaceuticals with electron donor properties (ω^+^ < 1.5) similar to dopamine and related neurotransmitters, presented in alphabetical order.

Name	ω^+^	ω^−^	Mechanism of action
5-OH-DPAT	0.74	4.10	D_2_ and D_3_ receptor full agonist
6-Br-APB	1.05	4.58	D_1_ full agonist
7-OH-DPAT	1.03	4.52	Selective D_3_ full agonist
7-OH-PIPAT	1.04	4.53	Selective D_3_ full agonist
A-412997	1.38	5.20	Selective D_4_ full agonist
A-77636	0.75	4.12	Selective D_1_ full agonist
A-86929	1.16	4.63	D_1_, D_2_ and D_5_ full agonist
Amfetamine	1.00	4.82	Dopaminergic stimulant, agonist-binding
Aplindore	1.07	4.47	Partial D_2_ agonist
Aptazapine	1.00	4.33	Dopamine antagonist
Aripiprazole	1.03	4.48	D_2_ partial agonist
Asenapine	1.03	4.77	D_1_, D_2_, D_3_ and D_4_ antagonist
Batanopride	1.34	4.95	D_2_ antagonist
BL-1020	1.38	4.68	D_2_ antagonist
Blonanserin	1.28	4.81	D_2_ and D_3_ antagonist
Brasofensine	1.21	5.2	Antidepressant
Brilaroxazine	1.19	4.67	D_2_, D_3_ and D_4_ partial agonist
Bromopride	1.45	5.18	D_2_ antagonist
Cabergoline	1.12	4.46	D_1_ and D_5_ full agonist and D_2_, D_3_ and D_4_ partial agonist
Cariprazine	1.24	4.83	D_2_ and D_3_ partial agonist
Chanoclavine I	1.11	4.43	Dopamine agonist
Chlorpromazine	1.37	4.69	D_1_, D_2_, D_3_ and D_5_ antagonist
*cis*8-OH-PBZI	1.05	4.57	D_3_ selective full agonist
Cyclindole	1.02	4.27	D_2_ antagonist
Desipramine	1.09	4.64	Antidepressant
Diethazine	1.18	4.44	Dopamine antagonist
Dihydrexidine	1.17	4.62	D_1_ and D_2_ agonist
Dihydroergocornine	1.10	4.43	D_1_ and D_2_ antagonist
Dihydroergocristine	1.11	4.43	Dopamine partial agonist
Dihydroergocryptine	1.11	4.45	D_2_ full agonist and D_1_ and D_3_ partial agonist
Dihydroergotamine	1.12	4.45	Dopaminergic ligand
Dinapsoline	1.11	4.62	Selective D_5_ full agonist
Dixyrazine	1.04	4.26	Dopamine antagonist
Dosulepin	1.43	5.02	Antidepressant
Ecopipam	1.21	4.91	D_1_ and D_5_ antagonist
Enciprazine	0.61	3.73	Antipsychotic and anxiolytic
Epicriptine	1.09	4.41	D_2_ full agonist and D_1_ and D_3_ partial agonist
Etoperidone	1.14	4.73	Weak dopamine antagonist
Fenoldopam	1.14	4.71	Selective D_1_ and D_5_ full agonist
Flibanserin	1.40	5.08	Selective D_4_ partial agonist
Flucindole	1.10	4.51	D_2_ antagonist
Gevotroline	1.24	4.75	D_2_ antagonist
Hordenine	0.71	4.05	D_2_ agonist
Imipramine	0.94	4.17	Antidepressant
Lergotrile	1.14	4.55	Dopamine agonist
Levomepromazine	1.09	4.25	D_2_ antagonist
Lodiperone	1.43	5.12	Dopamine antagonist
Mafoprazine	0.97	4.35	D_2_ antagonist
Mazapertine	1.51	5.12	D_2_ antagonist
Mequitazine	1.08	4.27	Dopamine antagonist
Mesulergine	1.14	4.44	D_2_ partial agonist
Methylphenidate	1.15	5.15	D_2_ ligand
Metoclopramide	1.27	4.86	D_2_ antagonist
Mirtazapine	1.31	4.80	Dopamine antagonist
Nortriptyline	1.37	5.13	Antidepressant
Pardoprunox	0.95	4.44	D_2_ and D_3_ partial agonist
PD-128,907	1.23	4.76	An experimental, selective D_2_ and D_3_ agonist
Perfenazine	1.29	4.65	D_2_ antagonist
Pergolide	1.07	4.37	Dopaminergic full agonist
PF-219061	1.12	4.82	Selective D_3_ agonist
PF-592379	1.35	5.04	Selective D_3_ agonist
Pimozide	0.98	4.41	D_2_ and D_3_ antagonist
Pramipexole	0.77	3.97	D_2_, D_3_ and D_4_ full agonist
Prochlorperazine	1.35	4.63	D_1_ and D_2_ antagonist
Promethazine	1.14	4.47	Dopamine antagonist
Quinagolide	0.88	4.32	D_1_ and D_2_ full agonist
Quinpirole	0.53	3.87	D_2_ and D_3_ full agonist
RDS-127	0.92	4.38	Selective D_2_ agonist
Remoxipride	1.46	5.33	D_2_, D_3_ and D_4_ antagonist
Ropinirole	1.09	4.68	D_2_, D_3_ and D_4_ agonist
Rotigotine	0.71	4.04	D_1_, D_2_, D_3_, D_4_ and D_5_ agonist
S-14297	1.05	4.44	Dopamine antagonist
SCH-23390	1.23	4.96	Selective D_1_ and D_5_ antagonist
Sertindole	1.39	4.90	D_2_ antagonist
SKF-38393	1.10	4.58	D_1_ and D_5_ partial agonist
SKF-77434	0.97	4.38	D_1_ partial agonist
SKF-81297	1.12	4.69	D_1_ full agonist
SKF-82958	1.05	4.58	A D_1_ full agonist
SKF-83959	1.06	4.59	D_1_ full agonist
SKF-89145	1.14	4.67	Selective D_1_ agonist
Spiroxatrine	0.92	4.21	Dopamine antagonist
Stepholidine	0.97	4.37	Dopamine antagonist
Sumanirole	1.01	4.50	Selective D_2_ full agonist
Talipexole	0.80	4.04	D_2_, D_3_ and D_4_ full agonist
Thiethylperazine	1.05	4.20	D_1_, D_2_ and D_4_ antagonist
Thioridazine	1.03	4.20	D_1_ and D_2_ antagonist
Trepipam	0.93	4.61	D_1_ agonist
Yohimbine	1.14	4.54	D_2_ and D_3_ antagonist
Zelandopam	0.97	4.41	A selective D_1_ agonist
Zetidoline	1.09	4.71	D_2_ antagonist
Zoloperone	1.44	5.11	Very weak dopamine antagonist

**Table 5:** Pharmaceuticals with electron acceptor properties (ω^+^ > 1.5), presented in alphabetical order.

Name	ω^+^	ω^−^	Mechanism of action
Abaperidone	2.55	6.94	D_2_ antagonist
Aceperone	2.51	6.99	Dopamine antagonist
Acepromazine	3.17	6.97	Dopamine antagonist
Acetophenazine	3.24	7.00	D_1_ and D_2_ antagonist
Alentemol	1.83	5.49	Selective D_2S_ agonist
Alizapride	2.59	6.87	D_2_ antagonist
Amiperone	2.60	7.04	Dopamine antagonist
Amisulpride	1.56	5.41	D_2S_, D_2L_ and D_3_ antagonist
Amoxapine	2.21	6.17	D_1_ and D_2_ antagonist
Apomorphine	1.77	5.55	D_1_ and D_2_ full agonist
Aporphine	1.86	5.79	D_1_ and D_2_ antagonist
Azabuperone	3.12	7.42	Dopamine antagonist
Azaperone	3.04	7.19	Dopamine antagonist
Benperidol	2.71	6.78	D_2_ antagonist
Bifeprunox	1.66	5.50	Weak D_2_ partial agonist
Biriperone	3.08	6.93	Dopamine antagonist
Boldine	1.71	5.31	Dopamine antagonist
Brexpiprazole	2.32	6.03	D_2_ partial agonist
Bromocryptine	2.04	5.79	D_1_, D_2_, D_3_ and D_5_ agonist and D_4_ antagonist
Bromperidol	2.51	6.99	Dopamine antagonist
Bulbocapnine	1.73	5.47	Dopamine antagonist
Buspirone	1.75	5.75	Weak D_2_ antagonist
Carperone	2.64	7.37	Dopamine antagonist
Carphenazine	3.09	6.87	D_1_, D_2_ and D_5_ antagonist
Chlorprothixene	1.96	5.74	D_1_, D_2_, D_3_ antagonist
Cicarperone	2.73	7.48	Dopamine antagonist
Cinuperone	2.31	6.09	D_2_ antagonist
Cloroperone	2.65	7.33	Dopamine antagonist
Clotiapine	1.99	5.86	Dopamine antagonist
Clozapine	2.04	5.79	D_1_, D_2_, D_3_ and D_4_ antagonist
Declenperone	2.77	6.86	Dopamine antagonist
Droperidol	2.72	6.82	D_2_ antagonist
Ergocornine	2.03	5.69	Dopamine agonist
α-Ergocryptine	1.97	5.61	Dopamine agonist
β-Ergocryptine	1.88	5.49	Dopamine agonist
Ergometrine	1.95	5.58	Dopamine agonist
α-Ergosine	1.90	5.53	Dopamine agonist
β-Ergosine	1.91	5.53	Dopamine agonist
Ergotamine	2.06	5.74	Dopamine agonist
Fananserin	2.94	7.06	D_4_ antagonist
Flufenazine	1.67	5.11	D_1_ and D_2_ antagonist
Flumezapine	1.75	5.33	Dopamine agonist
Flupenthixol	1.99	5.81	D_1_ and D_2_, antagonist
Fluperlapine	1.71	5.45	Dopamine antagonist
Glaucine	1.8	5.64	D_1_ and D_5_ antagonist
Haloperidol	2.51	6.99	D_1_ and D_2_ antagonist and a D_3_ and D_4_ inverse agonist
Homopipramol	5.87	2.15	Antidepressant with some antipsychotic effects
Iloperidone	2.40	6.66	Dopamine antagonist
Lenperone	2.49	7.14	Dopamine antagonist
Lisuride	1.80	5.40	D_2_, D_3_ and D_4_ full agonist, and D_1_ and D_5_ antagonist
Loxapine	2.20	6.14	D_1_ and D_2_ antagonist
Lumateperone	3.03	6.68	D_2S_ and D_2L_ partial agonist
Lurasidone	1.81	5.69	D_2_ antagonist
Melperone	2.46	7.10	D_2_ antagonist
Mesoridazine	1.63	5.17	D_2_ antagonist
Metopimazine	2.22	5.90	Dopamine antagonist
Metrenperone	2.63	6.72	Dopamine antagonist
Minaprine	1.93	5.85	D_1_ and D_2_ agonist
Moperone	2.81	7.26	A D_2_ antagonist
Nafadotride	3.01	7.27	D_3_ and D_2_ antagonist
Nemonapride	1.59	5.25	D_2_, D_3_ and D_4_ antagonist
Nonaperone	2.45	7.09	Dopamine antagonist
Norclozapine	2.08	5.83	Dopamine antagonist
Nuciferine	1.82	5.72	Dopamine weak antagonist
Ocaperidone	2.43	6.45	Dopamine antagonist
Olanzapine	1.72	5.27	D_1_, D_2_, D_3_, D_4_ and D_5_ antagonist
OSU-6162	1.77	6.19	D_2_ partial agonist
Paliperidone	1.78	5.89	D_1_, D_2_, D_3_ and D_4_ antagonist
PD-168,077	2.16	6.28	Selective D_4_ full agonist
Pentiapine	1.68	5.61	Dopamine antagonist
Perospirone	1.81	5.70	D_2_, D_3_ and D_4_ antagonist
Pimethixene	1.65	5.36	Dopamine antagonist
Pipamperone	2.62	6.83	D_4_ and D_2_ antagonist
Pipotiazine	2.07	5.65	D_1_ and D_2_ antagonist
Piribedil	1.77	5.61	D_2_ and D_3_ agonist
Prideperone	2.03	6.33	Dopamine antagonist
Primaperone	2.46	7.10	Dopamine antagonist
Propiomazine	3.03	6.88	Dopamine antagonist
Propyperone	3.33	7.37	Dopamine antagonist
Pukateine	1.76	5.52	Dopamine antagonist
Quetiapine	1.88	5.72	D_1_ and D_2_ antagonist
Quinelorane	1.66	5.58	D_2_ and D_3_ agonist
Raclopride	2.40	6.66	D_2_ and D_3_ antagonist
Rilapine	3.02	7.06	Dopamine antagonist
Risperidone	1.54	5.51	D_1_, D_2_, D_3_ and D_4_ antagonist
Ro10-5824	1.61	5.49	Selective D_4_ partial agonist
Roxindole	1.6	5.09	D_2S_, D_3_ and D_4_ antagonist
Roxoperone	2.45	7.09	Dopamine antagonist
Sarizotan	1.94	5.89	D_2_ antagonist
Setoperone	2.69	6.98	Dopamine antagonist
Spiperone	3.00	7.01	D_2_, D_3_ and D_4_ antagonist
Sulpiride	2.05	6.40	D_2_ and D_3_ antagonist
Tefluthixol	1.59	5.39	Dopamine antagonist
Tenilapine	3.25	7.57	Dopamine antagonist
Tetrabenazine	1.65	5.52	D_2_ ligand
Thiothixene	2.18	6.10	D_1_ and D_2_ antagonist
Tiapride	1.90	6.22	D_2_ and D_3_ and D_4_ antagonist
Timiperone	3.10	7.12	Dopamine antagonist
Tiospirone	1.81	5.70	Dopamine antagonist
Trifluoperazine	1.66	5.12	D_2_ antagonist
Trifluperidol	2.46	7.10	D_2_, D_3_ and D_4_ antagonist
UH-232	1.91	5.88	D_2_ antagonist and D_3_ partial agonist
Veralipride	2.28	6.73	Dopamine antagonist
Vilazodone	2.46	6.41	D_2_ weak agonist
Ziprasidone	1.81	5.70	D_2_, D_3_ and D_4_ antagonist
Zuclopenthixol	2.00	5.81	D_1_, D_2_ and D_5_ antagonist

One purpose of antipsychotic treatment is to minimize schizophrenia symptoms, which are caused by a deep imbalance in the dopaminergic system. Reported physiological mechanisms of schizophrenia demonstrate an excess of dopamine activity (direct or indirect) in certain regions of the brain, and little dopamine activity in other regions. We use our information to postulate that electron donors could be useful for modulating schizophrenia symptoms related to little dopamine activity as well as Parkinson’s disease and electron acceptors may be useful for controlling psychosis associated with an excess of dopamine activity as well as Huntington’s disease. Our findings indicate that electron acceptors bind to dopamine receptors and block or inactivate them. Contrarily, agonists interact and donate electrons, thus activating the receptor in a similar way to dopamine.

The drugs reported here were classified in the literature as agonists or antagonists. Additionally, electrochemical signaling in cells is an essential process in humans, indicating that electron transfer may be related to the functionality of the molecules that control psychosis. Our results agree with this theory and thus, it is in accordance with the currently believed molecular action mechanism of these drugs. Therefore, we corroborate previously reported postulations with quantum chemistry calculations, and also propose new information for this group of antipsychotic drugs.

The main idea of this investigation was to compare intrinsic properties (electron donor–acceptor) between the drugs and neurotransmitters. These intrinsic properties of the molecules are not always in agreement with the conventional classification of agonists and antagonists, specifically for those molecules of Family II that are classified experimentally as “partial” or “weak” agonists/antagonists. The new information reported in this study permits us to define these molecules as "similar to" or "different from" the neurotransmitters.

The design of drugs for specific treatments is very demanding. After chemical synthesis and all characterizations have been accomplished, it is necessary to carry out biological tests on the drugs to determine their efficacy, and also in this specific case to define whether they are conventional agonists or antagonists of dopamine or other neurotransmitters. There are many dopaminergic agents available, which vary in terms of effectiveness and side effects, and no single treatment works for all patients. When it is necessary to change medications for specific patients, it is no easy task to decide which medication will help control symptoms. The perception that emerges from this dilemma is that along with the experimental determinations and biological tests, it is possible to do quantum chemical calculations on the molecules in order to obtain more information about their inherent reactivity and susceptibility for binding to receptors. All this information together, including the comparison of these intrinsic chemical properties, should help medical doctors define the most suitable medication for each individual patient.

Notably, in this analysis we do not include dopamine receptors in the form of G-Protein-Coupled Receptors (GPCRs). This is because the principal aim of this investigation was to report information of the dopaminergic agents based on theoretical Density Functional Theory response functions, related to the electron transfer process. Previously^45^ it was reported that drugs are like light bulbs and receptors (GPCR proteins) resemble the sockets of a light bulb. Certain light bulb characteristics are independent of the sockets (for example, light bulbs can have different colors or voltage); in the same way that electron transfer properties of dopaminergic agents are independent of the receptors. This analogy is helpful in explaining the relevance of this information. All of these dopaminergic agents, ordered according to this new information, are reported in Tables 3 and 4. We also include Table 1S as supporting information with all the information reported until now about these drugs. We hope this information will be useful for better and rational treatment of psychosis.

## Conclusions

In this study, new information of 217 antipsychotics is presented based on the theoretical response functions related to the electron transfer process. In order to bind to dopamine receptors and inactivate them, molecules should be electron acceptors. Contrarily, agonists donate electrons and activate them, as dopamine does.

As reported previously, clinical use of these drugs is based on their classification as agonists or antagonists, and many times these classifications (based on experiments with animals) is not precise and is insufficient. For this reason, we hope that this new and more rational information will be functional as a guide in the clinical use of the drugs, improving treatment of psychosis, Parkinson’s disease and Huntington’s disease. This research provides new information concerning intrinsic properties of dopaminergic agents, which may be apt for their classification, once affinities for other receptors and biological effects have been taken into account.

## Methods

From the databases UniProt^50^, DrugBank 5.0^51^, Guide to Pharmacology^52^ and Inxight: Drugs^53^ pharmaceuticals with dopamine receptor affinity used as antipsychotics were selected for this study, particularly focusing on drugs used to treat psychosis. In total 217 (86 molecules categorized as agonists and 131 molecules classified as antagonists) compounds (Tables 1, 2) were selected and analyzed applying Density Functional Theory (DFT) calculations.

Gaussian09 was used for all electronic calculations^54^. Initial structures were taken from PubChem^55^ when available or several initial structures were used for the optimization*.* Geometry optimizations without symmetry constraints were implemented at M06/6–311 + G(2d,p) level of theory^56–59^, while applying the continuum solvation model density (SMD) with water, in order to mimic a polar environment^60^. M06 is one of the hybrid exchange correlation functional designed for main group thermochemistry. This functional has 27% of exact exchange; for the systems studied in this investigation higher percent is not required. Since negative ions are calculated, a triple-ζ basis set was used with diffuse and polarized functions*.* Harmonic analyses were calculated to verify local minima (zero imaginary frequencies). We considered protonated states of all drugs following the available experimental evidence. All molecular data of the optimized structures are available on request.

The response functions that we used in this investigation are the electro-donating (ω^−^) and electro-accepting (ω^+^) powers, previously reported by Gázquez et al*.*^61,62^. These authors defined the propensity to donate charge or ω^−^ (1) as follows:

1 $$\upomega ^{-} = \left( {3{\text{I}} + {\text{A}}} \right)^{2} /16\left( {{\text{I}} - {\text{A}}} \right) $$

whereas the propensity to accept charge or ω^+^ (2) is defined as

2 $$\upomega ^{ + } = \left( {{\text{I}} + 3{\text{A}}} \right)^{2} /16\left( {{\text{I}} - {\text{A}}} \right) $$

**Figure 5:**
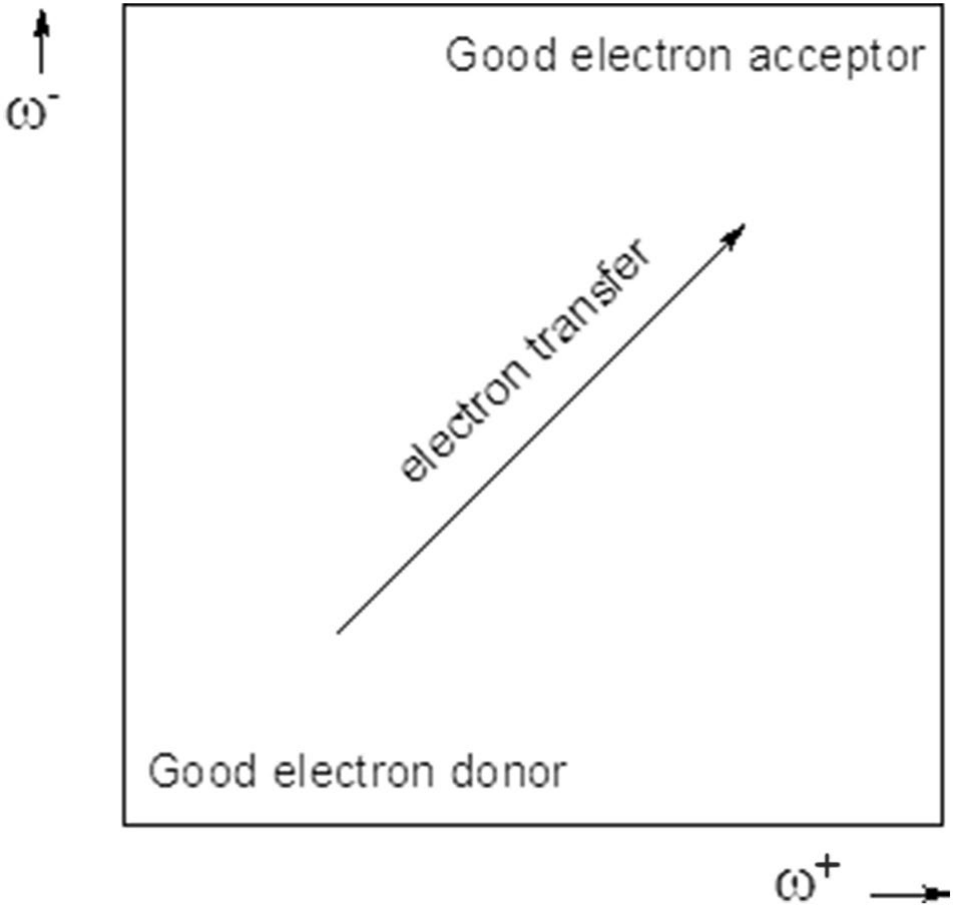
Donor–acceptor map (DAM).

I and A are vertical ionization energy and vertical electron affinity, respectively. Note that in ω^−^ the ionization energy has a higher weight in the equation and in ω^+^ electron affinity, which is in accordance with chemical intuition. Lower values of ω^−^ imply greater capacity for donating charge. Higher values of ω^+^ imply greater capacity for accepting charge. In contrast to I and A, ω^−^ and ω^+^ refer to charge transfers, not necessarily from one electron. This definition is based on a simple charge transfer model expressed in terms of chemical potential and hardness. The Donor–Acceptor Map previously defined^49^ is a useful graphical tool that has been used successfully in many different chemical systems^63–65^. We have plotted ω^−^ and ω^+^ (Fig. 5) on this map, enabling us to classify substances as either electron donors or acceptors. Electrons are transferred from good donor systems (down to the left of the map) to good electron acceptor systems (up to the right of the map). In order to analyze electron-donor acceptor properties, vertical ionization energy (I) and vertical electron affinity (A) were obtained from single point calculations of the corresponding cationic and anionic molecules, using the optimized structure of the neutrals. The same level of theory was used for all computations.

## Supplementary Information

Supplementary Information.
