# Supplementary material for: New information of dopaminergic agents based on quantum chemistry calculations
Source: Sci Rep. 2020 Dec 9;10:21581. doi: 10.1038/s41598-020-78446-4 (PMC7725812; doi:10.1038/s41598-020-78446-4)
Supplement: Supplementary file 1 — Supplementary Information. [file 41598_2020_78446_MOESM1_ESM.docx]

**New information of dopaminergic agents based on quantum chemistry calculations**

**Guillermo Goode-Romero^1^ · Ulrika Winnberg^2^ · Laura Domínguez^1^ · Ilich A. Ibarra^3^ · Rubicelia Vargas^4^ · Elisabeth Winnberg^5^ · Ana Martínez^6^**

**Ana Martínez (corresponding author)**

[martina@unam.mx](mailto:martina@unam.mx)

**Guillermo Goode-Romero (corresponding author)**

[guillermo_david_goode@comunidad.unam.mx](mailto:guillermo_david_goode@comunidad.unam.mx)

^1^ Departamento de Fisicoquímica, Facultad de Química, Universidad Nacional Autónoma de México, Circuito Exterior SN, Ciudad Universitaria, CP 04510, CDMX, México

^2^ Departamento Académico de Ingeniería Industrial y Operaciones, Instituto Tecnológico Autónomo de México, Río, Hondo 1, Altavista, Álvaro Obregón, CP 01080 Ciudad de México, CDMX, México

^3^ Laboratorio de Fisicoquímica y Reactividad de Superficies (LaFReS), Instituto de Investigaciones en Materiales, Universidad Nacional Autónoma de México, Circuito Exterior SN, Ciudad Universitaria, CP 04510, CDMX, México

^4^ Departamento de Química, División de Ciencias Básicas e Ingeniería, Universidad Autónoma Metropolitana-Iztapalapa, San Rafael Atlixco 186, Col. Vicentina, Iztapalapa. AP Postal 55-534, CP 09340, CDMX, México

^5^ Department of Health Care Sciences, Ersta Sköndal Bräcke University College, Stigbergsgatan 30, SE-116 28 Stockholm, Sweden

^6^ Departamento de Materiales de Baja Dimensionalidad, Instituto de Investigaciones en Materiales, Universidad Nacional Autónoma de México, Circuito Exterior SN, Ciudad Universitaria, CP 04510, CDMX, México. Phone (52) 55-5622-4596

Table 1S. Complete information of the molecules under study in alphabetical order.

| **Name** | **ω^-^** | **ω^+^** | **Structure** | **Conventional classification, mechanism of action and receptor targets** | **Family** | **Therapeutic indications** | |
| --- | --- | --- | --- | --- | --- | --- | --- |
| 5-OH-DPAT | 4.10 | 0.74 | 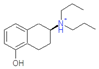 | Only the (*R*) enantiomer is selective agonist of D_2_ receptor. The (*S*) enantiomer is a weak partial agonist of both D_2_ and D_3_ [5] | I | Research | |
| 6-Br-APB | 4.58 | 1.05 | 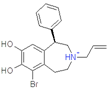 | Selective agonist of D_1_ | I | Research | |
| 7-OH-DPAT | 4.52 | 1.03 | 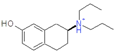 | Selective D_3_ full agonist [6,7] | I | Research for addiction to cocaine in animal models | |
| 7-OH-PIPAT | 4.53 | 1.04 | 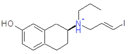 | Selective D_3_ agonist [8] | I | Research | |
| 8-OH-DPAT | 4.29 | 0.85 | 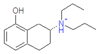 | 5HT_1A_ agonist | I | Research | |
| A412997 | 5.20 | 1.38 | 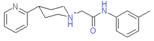 | Selective D_4_ agonist | I | Research | |
| A77636 | 4.12 | 0.75 | 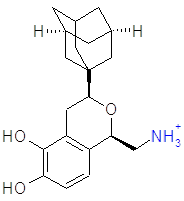 | Selective D_1_ agonist | I | Research | |
| A86929 | 4.63 | 1.16 | 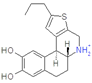 | A potent full D_1_, D_2_ and D_5_ agonist | I | Active metabolite of adrogolide.  Research in cocaine abuse and Parkinson’s disease | |
| Abaperidone | 6.94 | 2.55 | 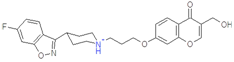 | Atypical antipsychotic.  D_2_ and 5HT_2A_ antagonist | I | Research for schizophrenia | |
| Aceperone | 6.99 | 2.51 | 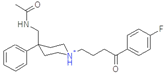 | Neuroleptic, typical antipsychotic.  α antagonist | I | Psychotic disorders | |
| Acepromazine | 6.97 | 3.17 | 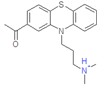 | Neuroleptic, typical antipsychotic.  Dopamine and muscarinic antagonist | I | Veterinary use as tranquilizer/sedative and antiemetic  Little use in human psychoses | |
| Acetophenazine | 7.00 | 3.24 | 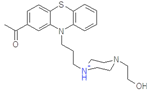 | Neuroleptic, typical antipsychotic.  D_1_, D_2_, SIGMAR1 and AR antagonist | I | Previously used in psychoses | |
| ACP-104 | 5.83 | 2.08 | 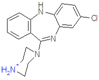 | Antipsychotic, metabolite of clozapine.  5HT_2A_ inverse agonist, D_2_ and D_3_ partial agonist and M_1_ and M_2_ allosteric activator | II | Schizophrenia | |
| Alentemol | 5.49 | 1.83 | 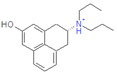 | Selective D_2_S (autoreceptor) agonist | II | Research with antipsychotic effects | |
| Alizapride | 6.87 | 2.59 | 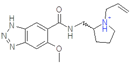 | Antiemetic and gastroprokinetic.  D_2_ antagonist | I | Nausea and emesis, including postoperative. | |
| Amiperone | 7.04 | 2.60 | 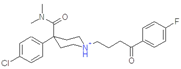 | Neuroleptic, typical antipsychotic | I | Research | |
| Amisulpride | 5.41 | 1.56 | 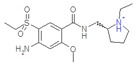 | Atypical antipsychotic and antidepressant.  D_2_S and presynaptic D_3_ agonist at low doses, D_2_L and postsynaptic D_3_ at high doses. Also 5HT_2A_ and 5HT_7_ antagonist | I | Schizophrenia and dysthymia | |
| Amoxapine | 6.17 | 2.21 | 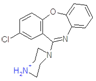 | Atypical antipsychotic and sedative antidepressant, metabolite of loxapine.  Antagonist of D_1_, D_2_, 5HT_1A_, 5HT_1B_, 5HT_2A_, 5HT_2C_, 5HT_3A_, 5HT_6_, 5HT_7_, α_1_, α_2_, NET, SERT, DAT and a GABA_A_ binder | I | Depressive disorder, psychoses, depressive phase of bipolar disorder | |
| (*S*)-Amphetamine | 4.82 | 1.00 | 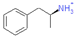 | Stimulant.  TAAR_1_, α, β, and D_2_^high^ agonist, DAT, SVAT and MAO inhibitor and NET, carbonic anhydrase 5A and carbonic anhydrase 7 activator | I | ADHD, narcolepsy | |
| Aptazapine | 4.33 | 1.00 | 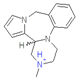 | Atypical antipsychotic and antidepressant.  Antagonist of α_2_, 5HT_2_ and H_1_ | II | Depressive disorder | |
| Aplindore | 4.47 | 1.07 | 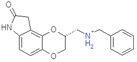 | Atypical antipsychotic.  D_2_ partial agonist | I | Schizophrenia. | |
| (*R*)-Apomorphine | 5.55 | 1.77 | 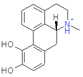 | Partial D_1_, D_2_ [9–11], D_3_, D_4_, D_5_, 5HT_1A_ and α_2A_ agonist. Also 5HT_2A_, 5HT_2B_, 5HT_2C_, α_1D_, α_2B_ and α_2C_ agonist, and TRPA1 activator | II | Parkinson’s disease, and is used to induce penile erection by inyection *in situ*. | |
| (*S*)-Apomorphine | 5.78 | 1.94 | 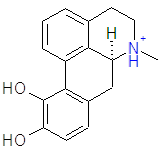 | D_1_ and D_2_ antagonist [9,10], and Nrf2 inductor | II | Research in amyotrophic lateral sclerosis | |
| (*R*)-Aporphine | 5.79 | 1.86 | 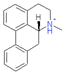 | D_1_, 5HT_7_ and 5HT_1A_ antagonist [12] | II | Research | |
| (*S*)-Aporphine | 5.80 | 1.47 | 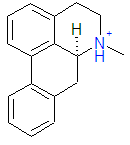 | Dopamine binder | II | Research | |
| Aripiprazole | 4.48 | 1.03 | 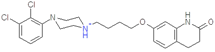 | Atypical antipsychotic and thymomodulator.  D_1_, D_2_, D_3_, D_4_, D_5_ and 5HT_1A_ partial agonist, 5HT_1B_, 5HT_1D_, 5HT_1E_, 5HT_2A_, 5HT_2C_, 5HT_7_, M_1_, M_2_, M_3_, M_4_, M_5_, α_1A_, α_1B_, α_2A_, α_2B_, α_2C_ and H_1_ antagonist and SERT and DAT modulator | I | Schizophrenia, bipolar disorders, depressive disorder, psychomotor agitation, Tourette’s syndrome, autistic spectrum related disorders | |
| Asenapine | 4.77 | 1.03 | 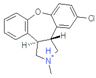 | Atypical antipsychotic.  D_1_, D_2_, D_3_, D_4_, 5HT_2A_, 5HT_2B_, 5HT_2C_, 5HT_5A_, 5HT_6_, 5HT_7_, α_1A_, α_2A_, α_2B_, α_2C_, β_1_, β_2_, H_1_, H_2_ antagonist, 5HT_1D_ full agonist and 5HT_1A_, 5HT_1B_ binder | II | Schizophrenia, bipolar disorders, posttraumatic stress disorder | |
| Azabuperone | 7.42 | 3.12 | 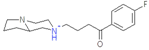 | Tranquilizer and neuroleptic, typical antipsychotic..  Dopamine antagonist | I | Under investigation | |
| Azaperone | 7.19 | 3.04 | 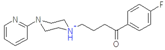 | Neuroleptic, typical antipsychotic.  Dopamine antagonist | I | Veterinary use for aggressive behavior | |
| Batanopride | 4.95 | 1.34 | 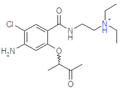 | Antiemetic and gastroprokinetic.  5HT_3_ inactivator | II | Nausea and emesis | |
| Benperidol | 6.78 | 2.71 | 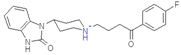 | Neuroleptic, typical antipsychotic.  D_2_, D_4_, H_1_, M, α_1_ antagonist | I | Schizophrenia | |
| Bicifadine | 4.56 | 0.93 | 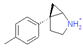 | Antinociceptive, stimulant antidepressant.  Inhibitor of NET, DAT, SERT | I | Chronic pain | |
| Bifeprunox | 5.50 | 1.66 | 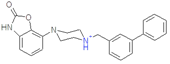 | Dopamine modulator.  Weak partial D_2_ agonist and 5HT_1A_ agonist | II | Depressive phase of bipolar disorders, schizophrenia, Parkinson’s disease | |
| Biriperone | 6.93 | 3.08 | 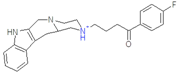 | Neuroleptic, typical antipsychotic.  D_2_ and 5HT_2A_ antagonist | I | Schizophrenia | |
| Blonanserin | 4.81 | 1.28 | 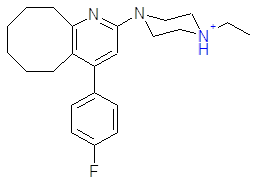 | Atypical antipsychotic.  D_2_, D_3_, 5HT_2A_ antagonist. | I | Schizophrenia | |
| (*R*)-Boldine | 5.31 | 1.71 | 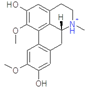 | Antagonist of α-adrenergic receptors | II | Research | |
| (*S*)-Boldine | 5.43 | 1.71 | 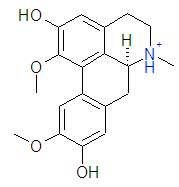 | D_1_ and D_2_ antagonist [11], AChE and telomerase inhibitor, and glial hemichannel blocker [13] | II | Research | |
| Brexpiprazole | 6.03 | 2.32 | 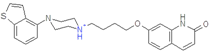 | Atypical antipsychotic and thymomodulator.  Partial D_2_, D_3_ and 5HT_1A_ agonist and 5HT_2A_, 5HT_2B_, 5HT_2C_, α_1B_ and α_2C_ antagonist | II, | Depressive disorder, schizophrenia | |
| Brasofensine | 5.20 | 1.21 | 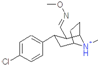 | Stimulant.  DAT inhibitor | I | Research in Parkinson’s disease and depressive disorders | |
| Brilaroxazine | 4.67 | 1.19 | 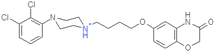 | Atypical antipsychotic.  Partial D_2_, D_3_ and D_4_ agonist | I | Investigated for schizophrenia and schizoaffective disorder | |
| Bromocryptine | 5.79 | 2.04 | 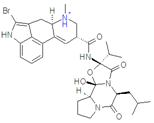 | Antiparkinsonian.  D_1_, D_2_, D_3_, D_5_, 5HT_1A_, 5HT_1B_, 5HT_1D_, 5HT_2A_, 5HT_2B_, 5HT_2C_, 5HT_7_, α_1D_, α_2A_, α_2B_ and α_2C_ agonist, α_1A_ and α_1B_ weak agonist, and D_4_ antagonist | II | Galactorrhea, amenorrhea, migraine and tumor-promoting prolactin-dependent breast-associated diseases. | |
| Bromopride | 5.18 | 1.45 | 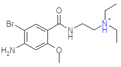 | Antiemetic and gastroprokinetic.  D_2_ antagonist and ChE inhibitor | II | Dyspepsia, nausea and emesis, and gastroesophageal reflux disease | |
| Bromperidol | 6.99 | 2.51 | 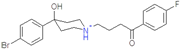 | Neuroleptic, typical antipsychotic.  D_2_ antagonist | I | Schizophrenia | |
| (*R*)-Bulbocapnine | 5.47 | 1.73 | 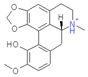 | D_1_ and D_2_ antagonist [11], AChE and tyrosine hydroxylase inhibitor, and amyloid-beta fibril formation [14] | II | Research | |
| (*S*)-Bulbocapnine | 5.54 | 1.77 | 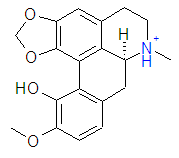 | D_1_ and D_2_ antagonist | II | Research | |
| Buspirone | 5.75 | 1.75 | 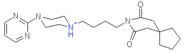 | Atypical anxiolytic.  Partial 5HT_1A_ and 5HT_7_ agonist, D_2_, D_3_ and D_4_ antagonist, and weak α_1A_ agonist | I | Anxiety disorders | |
| Cabergoline | 4.46 | 1.12 | 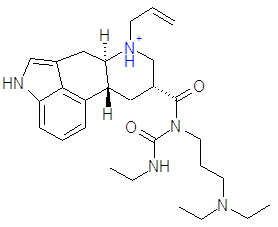 | Antiparkinsonian.  D_2_, D_3_ and 5HT_2B_ agonist | I | Hyperprolactinemia, prolactinoma, galactorrhea, acromegaly, Parkinson’s disease | |
| Cariprazine | 4.83 | 1.24 | 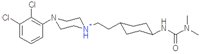 | Atypical antipsychotic.  Partial D_2_, D_3_ and 5HT_1A_ gonist, and 5HT_2A_, 5HT_2B_ and H_1_ antagonist | I | Schizophrenia and bipolar I disorder | |
| Carperone | 7.37 | 2.64 | 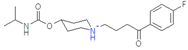 | Neuroleptic, typical antipsychotic.  Dopamine antagonist | I | Psychosis | |
| Carphenazine | 6.87 | 3.09 | 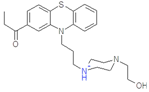 | Neuroleptic, typical antipsychotic.  Antagonist of D_1_, D_2_ and D_5_ | I | Schizophrenia | |
| Chanoclavine I | 4.43 | 1.11 | 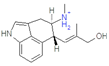 | Dopamine binder | I | Research | |
| Chlorpromazine | 4.69 | 1.37 | 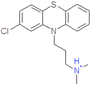 | Neuroleptic, typical antipsychotic.  5HT_2A_ inverse agonist and D_1_, D_2_, D_3_, D_5_, 5HT_1A_, 5HT_2C_, α_1_, α_2_, H_1_, M_1_ and M_3_ antagonist | II | Schizophrenia, bipolar disorders, migraine, nausea and emesis, acute intermittent porphyria | |
| Chlorprothixene | 5.74 | 1.96 | 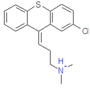 | Neuroleptic, typical antipsychotic.  D_1_, D_2_, D_3_, 5HT_2A_, 5HT_2B_, 5HT_2C_, H_1_, M_1_, M_2_, M_3_, M_4_ and M_5_ antagonist | I | Psychotic disorders | |
| Cicarperone | 7.48 | 2.73 | 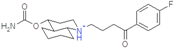 | Neuroleptic, anesthetic.  Ion channels blocker | I | Local anesthetic and cardioprotective in animals | |
| Cinitapride | 9.22 | 4.59 | 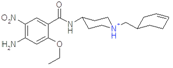 | Gastroprokinetic.  5HT_2A_ antagonist and 5HT_1A_ and 5HT_4_ agonist | I | Dyspepsia, nausea and emesis, and gastroesophageal reflux disease, peptic ulcer | |
| Cinuperone | 6.09 | 2.31 | 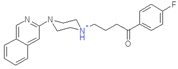 | Neuroleptic, typical antipsychotic.  D_2_, α_1A_ and SIGMAR1 antagonist | I | Psychotic diseases | |
| *cis*8OH-PBZI | 4.57 | 1.05 | 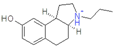 | Selective D_3_ agonist [15] | I | Research | |
| Cisapride | 5.26 | 1.57 | 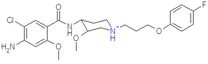 | Gastroprokinetic.  5HT_2A,_ 5HT_3A,_ 5HT_4_ agonist | I | Dyspepsia, nausea and emesis, and gastroesophageal reflux disease | |
| Clebopride | 4.76 | 1.21 | 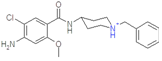 | Gastroprokinetic.  D_2_ antagonist | II | Nausea and emesis | |
| Cloroperone | 7.33 | 2.65 | 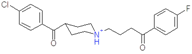 | Neuroleptic, typical antipsychotic.  5HT2 binder | I | Investigated for anxiety, emesis and psychotic diseases | |
| Clotiapine | 5.86 | 1.99 | 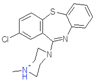 | Atypical antipsychotic.  GABA_A_ benzodiazepine site inactivator | I | Research | |
| Clozapine | 5.79 | 2.04 | 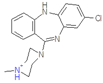 | Atypical antipsychotic.  5HT_2A_, 5HT_2C_, 5HT_6_ and 5HT_7_ inverse agonist, D_1_, D_2_, D_3_, D_4_, α_1A_, α_1B_, α_2A_, α_2B_, α_2C_, H_1_, H_3_ and H_4_ antagonist, 5HT3 inactivator, 5HT_1A_, 5HT_1B_, 5HT_1D_ and 5HT_1F_ full agonist, M_1_ allosteric activator and K_ir_3.2 blocker. Also a DNMT1 downregulator. | I | Schizophrenia, bipolar disorders, intermittent explosive disorder and posttraumatic stress disorder | |
| Cyclindole | 4.27 | 1.02 | 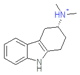 | Atypical antipsychotic.  D_2_ antagonist | II | Research with antidepressant and neuroleptic properties | |
| Declenperone | 6.86 | 2.77 | 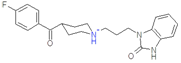 | Neuroleptic.  5HT_2A_ antagonist | I | Veterinary use as sedative | |
| Desipramine | 4.64 | 1.09 | 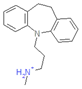 | Tricyclic antidepressant.  5HT_2A_, α_1_, H_1_ and muscarinic antagonist, NET and SERT inactivator, and K_ir_3.2 and K_ir_3.4 blocker | II | Depressive disorder, anxiety disorders, chronic pain disorder, fibromyalgia and migraine prophylaxis | |
| Diethazine | 4.44 | 1.18 | 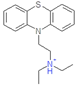 | Neuroleptic, typical antipsychotic.  Dopamine, muscarinic and H_1_ antagonist | II | Parkinson’s disease and allergic manifestations | |
| Dihydrexidine | 4.62 | 1.17 | 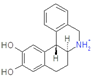 | Moderately selective D_1_ and D_5_ agonist | I | Investigated for Parkinson’s disease and for the treatment of cognitive impairments of schizophrenia and schizotypal disorder | |
| Dihydroergocornine | 4.43 | 1.10 | 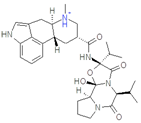 | D_1_ and D_2_ antagonist, and weak serotonin, α and β binder | I | Adjuvant in vascular dementia and age-related cognitive impairment, as well as to aid in recovery after stroke | |
| Dihydroergocristine | 4.43 | 1.11 | 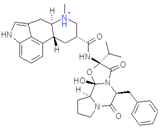 | Partial dopamine agonist, weak adrenergic agonist and 5HT_6_ antagonist | I | Adjuvant in vascular dementia and age-related cognitive impairment, as well as to aid in recovery after stroke | |
| Dihydroergocryptine | 4.45 | 1.11 | 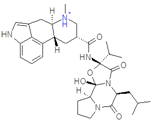 | D_2_ and biased 5HT_2B_ agonist, D_1_ and D_3_ partial agonist, and 5HT_7_ antagonist | I, | Adjuvant in vascular dementia and age-related cognitive impairment, as well as to aid in recovery after stroke | |
| Dihydroergotamine | 4.45 | 1.12 | 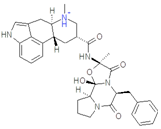 | D_2_, D_3_, 5HT_1A_, 5HT_1B_, 5HT_1D_, 5HT_1F_, 5HT_2A_, 5HT_2B_, 5HT_2C_, α_2A_ agonist, and 5HT_6_ and 5HT_7_ antagonist | I | Prophylaxis of migraine and Raynaud’s disease, acute treatment for status migrainosus | |
| Dinapsoline | 4.62 | 1.11 | 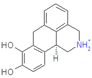 | Selective D_5_ agonist. | I | Research | |
| Dixyrazine | 4.26 | 1.04 | 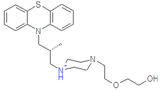 | Neuroleptic, typical antipsychotic.  Dopamine antagonist | II | Psychotic disorders, nausea and emesis | |
| Domperidone | 4.41 | 0.98 | 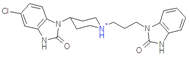 | Gastroprokinetic.  Peripherally selective D_2_ and D_3_ antagonist | II | Gastroparesia, gastroesophageal reflux disease | |
| Dosulepin | 5.02 | 1.43 | 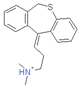 | Tricyclic antidepressant.  5HT_1A_, 5HT_2A_, α_1_, α_2_, H_1_, M_1_, M_2_, M_3_, M_4_ and M_5_ antagonist, and DAT, NET and SERT inhibitor | II | Depressive disorder, neuropathic pain | |
| Droperidol | 6.82 | 2.72 | 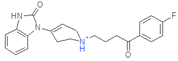 | Neuroleptic, typical antidepressant, anesthesia inducer.  D_2_, 5HT_2A_ and α_1A_ antagonist | I | Tranquilizer, and in nausea and emesis | |
| Ecopipam | 4.91 | 1.21 | 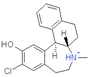 | D_1_ and D_5_ antagonist | II | In trials studying the treatment of Tourette's Syndrome, Lesch-Nyhan Disease, Pathological Gambling and Self-injurious Behavior. | |
| Enciprazine | 3.73 | 0.61 | 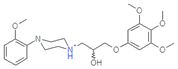 | Atypical anxiolytic.  Allosteric GABA_A_ activator | II | Anxiety disorders | |
| Epicryptine | 4.41 | 1.09 | 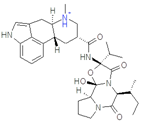 | Dopamine agonist | I | Prophylaxis of migraine | |
| Ergocornine | 5.69 | 2.03 | 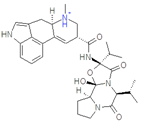 | Dopamine and serotonin agonist | II | Tumor-promoting prolactin-dependent breast-associated diseases | |
| α-Ergocryptine | 5.61 | 1.97 | 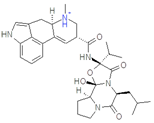 | D_2_ agonist and 5HT_6_ antagonist | II | Research in ocular hypertension | |
| β-Ergocryptine | 5.49 | 1.88 | 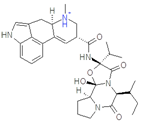 | D_5_ agonist [16] | II | Adjuvant in vascular dementia and age-related cognitive impairment, as well as to aid in recovery after stroke | |
| α-Ergosine | 5.53 | 1.90 | 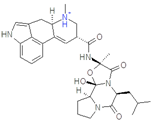 | D_1_ agonist, and 5HT_1_ and α_2_ antagonist | II | Adjuvant in vascular dementia and age-related cognitive impairment, as well as to aid in recovery after stroke | |
| β-Ergosine | 5.53 | 1.91 | 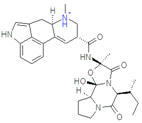 | Dopaminergic and adrenergic ligand | II | Adjuvant in vascular dementia and age-related cognitive impairment, as well as to aid in recovery after stroke | |
| Ergometrine | 5.58 | 1.95 | 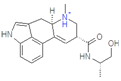 | Oxytocic.  α_1A_ agonist and partial serotonin agonist | II | Postpartum hemorrhage and post-abortion hemorrhage | |
| Ergotamine | 5.74 | 2.06 | 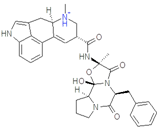 | Vasoconstrictor.  D_1_, D_2_, 5HT_1A_, 5HT_1B_, 5HT_1D_, 5HT_1F_, 5HT_2A_, 5HT_2C_ agonist, partial α_1A_, α_1B_, α_1D_, α_2A_ and α_2B_ agonist, and NET inhibitor | II | Prophylaxis of migraine, cluster headache, ophthalmoplegic migraine and basilar migraine | |
| Etoperidone | 4.73 | 1.14 | 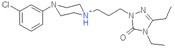 | Atypical antidepressant.  D_2_, 5HT_2A_, α_1_, α_2_ and muscarinic antagonist, and 5HT_2C_ agonist | II | Depressive disorder and Adjuvant in Parkinson’s disease | |
| Fananserin | 7.06 | 2.94 | 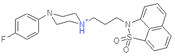 | Antipsychotic.  D_4_ and 5HT_2A_ antagonist | I | Investigated for the treatment of schizophrenia | |
| Fenoldopam | 4.71 | 1.14 | 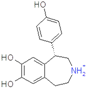 | Selective D_1_ and D_5_ agonist, and α_1A_, α_1B_, α_1D_, α_2A_, α_2B_ and α_2C_ antagonist | I | Severe hypertension and malignant hypertension | |
| Flibanserin | 5.08 | 1.40 | 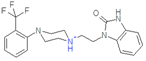 | Selective partial D_4_ agonist, 5HT_1A_ agonist and 5HT_2A_ antagonist | I | Treatment of pre-menopausal women with hypoactive sexual desire disorder | |
| Flucindole | 4.51 | 1.10 | 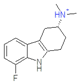 | Atypical antipsychotic.  D_2_ antagonist | II | Investigated for the treatment of psychotic disorders | |
| Flumezapine | 5.33 | 1.75 | 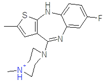 | Atypical antipsychotic.  D_2_ and serotonin antagonist | I | Investigated for the treatment of psychotic disorders | |
| Flupenthixol | 5.81 | 1.99 | 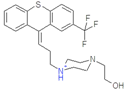 | Typical antipsychotic.  D_1_, D_2_, 5HT_2A_, α_1A_ and M_1_ antagonist | I | Psychotic disorders and depressive disorders | |
| Fluperlapine | 5.45 | 1.71 | 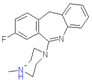 | Atypical antipsychotic.  5HT_2C_ inverse agonist | I | Research in psychotic disorders | |
| Fluphenazine | 5.11 | 1.67 | 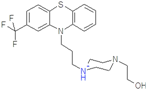 | Typical antipsychotic.  5HT_6_ and 5HT_7_ inverse agonist, D_1_, D_2_, D_5_, H_1_ and 5HT_2A_ antagonist and CaM blocker | I | Psychotic disorders | |
| Gevotroline | 4.75 | 1.24 | 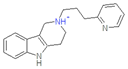 | Atypical antipsychotic.  D_2_, 5HT_2_ and SIGMAR1 antagonist | I | Research in psychotic disorders | |
| (*R*)-Glaucine | 5.64 | 1.80 | 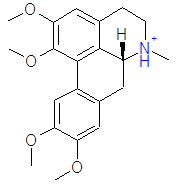 | Allosteric 5HT_2A_ activator, α_1_ antagonist, and PDE4 inhibitor | II | Research | |
| (*S*)-Glaucine | 5.64 | 1.80 | 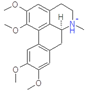 | Partial 5HT_2A_, 5HT_2B_ and 5HT_2C_ agonist, D_1_, D_5_ and α_1_ antagonist, and PDE4 inhibitor | II | Research | |
| Haloperidol | 6.99 | 2.51 | 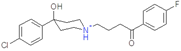 | Neuroleptic, typical antipsychotic.  D_3_ and D_4_ inverse agonist, D_1_, D_2_, 5HT_1A_, 5HT_2A_, 5HT_2C_, H_1_, M_3_ and SIGMA1 antagonist, and allosteric NMDAR inactivator | I | Schizophrenia, Tourette’s disease, intermittent explosive disorder, hyper-excitability and agitation, Huntington’s disease, delirium tremens, obsessive-compulsive disorder, severe disruptive behavior disorder | |
| Homopipramol | 5.87 | 2.15 | 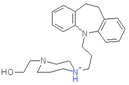 | Tricyclic antidepressant.  NET inhibitor | I | Research in depressive disorder | |
| Hordenine | 4.05 | 0.71 |  | TAAR1 and D_2_ agonist, and reversible MAO-B inhibitor | I | Research | |
| Iloperidone | 6.66 | 2.40 |  | Atypical antipsychotic.  D_1_, D_2_, D_3_, D_4_, 5HT_1A_, 5HT_2A_, 5HT_6_, 5HT_7_, α_1A_, α_2C_ and H_1_ antagonist | I | Psychotic disorders | |
| Imipramine | 4.17 | 0.94 |  | Tricyclic antidepressant.  5HT_2A_, 5HT_2C_, 5HT_7_, α_1A_, H_1_, M_1_, M_2_, M_3_, M_4_, M_5_ antagonist, SERT and NET inhibitor, and 5HT_1A_ agonist | II | Depressive disorders, chronic pain disorder, fibromyalgia, migraine | |
| Itopride | 5.27 | 1.55 |  | D2 antagonist, and AChE inhibitor | I | Gastroparesis, irritable bowel syndrome | |
| Lenperone | 7.14 | 2.49 |  | Neuroleptic, typical antipsychotic.  Dopamine and 5HT2 antagonist | I | Research in psychotic disorders | |
| Lergotrile | 4.55 | 1.14 |  | Dopamine agonist | I | Parkinson’s disease | |
| Levomepromazine | 4.25 | 1.09 |  | Neuroleptic, typical antipsychotic.  D_1_, D_2_, D_3_, D_4_, D_5_, 5HT_2A_, 5HT_2C_, α_1A_, α_1B_, α_1D_, α_2A_, α_2B_, α_2C_, H_1_, M_1_, M_2_, M_3_,  M_4_ and M_5_ antagonist | II | Schizophrenia, bipolar disorders, agitation, sleep initiation and maintenance disorder, chronic pain, nausea and emesis, phantom limb pain, post-herpetic neuralgia, senile psychosis, trigeminal neuralgia | |
| Lisuride | 5.40 | 1.80 |  | Antiparkinsonian.  5HT_1A_ and 5HT_7_ full agonist, partial D_2_, D_3_, D_4_, 5HT_1B_, 5HT_1D_, 5HT_2A_, 5HT_2C_, and 5HT_6_ agonist, weak D_1_ and D_5_ agonist, and 5HT_2B_, α_1A_, α_2A_, α_2B_ and α_2C_ antagonist | II | Parkinson’s disease, postencephalitic parkinsonism, amenorrhea, galactorrhea, acromegaly and tumor-promoting prolactin-dependent breast-associated diseases | |
| Lodiperone | 5.12 | 1.43 |  | Atypical antipsychotic.  Dopamine antagonist | II | Psychotic disorders | |
| Loxapine | 6.14 | 2.20 |  | Atypical antipsychotic and sedative antidepressant.  Antagonist of D_1_, D_2_, 5HT_2A_, 5HT_2C_, α_2_, H_1_ and M_1_ | I | Schizophrenia, acute agitation | |
| Lumateperone | 6.68 | 3.03 |  | Antipsychotic.  Partial D_2S_ and D_2L_ agonist | I | Schizophrenia, bipolar disorder | |
| Lurasidone | 5.69 | 1.81 |  | Atypical antipsychotic.  D_2_, 5HT_2A_, 5HT_7_ and α_2C_ antagonist and partial 5HT_1A_ agonist | I | Schizophrenia, bipolar disorders, depressive disorder, autistic spectrum disorders | |
| Mafoprazine | 4.35 | 0.97 |  | Psycholeptic, atypical antipsychotic.  D_2_ and α_1_ antagonist, and α_2_ agonist | II | Investigated for the treatment of psychotic disorders and aggressive behavior | |
| Mazapertine | 5.12 | 1.51 |  | Atypical antipsychotic.  D_2_, D_3_, α_1_ and 5HT_1A_ antagonist | II | Investigated for the treatment of schizophrenia | |
| Melperone | 7.10 | 2.46 |  | Typical antipsychotic.  Weak agonist of D_2_ | I | Psychomotor agitation, sleep disorders, confusion states | |
| Mequitazine | 4.27 | 1.08 |  | Neuroleptic.  H_1_ and muscarinic antagonist | II | Hay fever, allergic rhinitis, urticaria | |
| Mesoridazine | 5.17 | 1.63 |  | Neuroleptic, typical antipsychotic.  Antagonist of D_2_ and 5HT_2A_ | I | Schizophrenia, dementia, Korsakoff psychosis | |
| Mesulergine | 4.44 | 1.14 |  | Partial D_2_ agonist, 5HT_2A_ antagonist and 5HT_2C_ inverse agonist | I | Research in Parkinson’s disease | |
| Methylphenidate | 5.15 | 1.15 |  | Psychoanaleptic, stimulant.  DAT and NET inhibitor, 5HT_1A_ binder | I | ADHD, narcolepsy, psychomotor agitation | |
| Metoclopramide | 4.86 | 1.27 |  | Antiemetic gastroprokinetic.  D_2_, M_1_ and M_3_ antagonist, 5HT_4_ agonist and 5HT_3_ inactivator | II | Gastroesophageal reflux disease, nausea and emesis, hiccups, gastroparesis | |
| Metopimazine | 5.90 | 2.22 |  | Neuroleptic, typical antipsychotic.  D_2_, H_1_ and α_1_ antagonist | I | Agitation, nausea and emesis | |
| Metrenperone | 6.72 | 2.63 |  | Neuroleptic.  Serotonin antagonist | I | Veterinary use | |
| Minaprine | 5.85 | 1.93 |  | D_1_, D_2_ and M_1_ agonist, 5HT_2_ antagonist and MAO-A and AChE inhibitor | II | Research in depressive disorders | |
| Mindoperone | 7.20 | 3.43 |  | Dopamine antagonist | I | Research in psychotic disorders | |
| Mirtazapine | 4.80 | 1.31 |  | Psycholeptic, sedative antidepressant.  5HT_2A_, 5HT_2C_, 5HT_3A_, α_1_, α_2A_ and H_1_ antagonist and KOR agonist | II | Depressive disorder, dysthymia, insomnia, panic disorder, obsessive-compulsive disorder, posttraumatic disorder, sleep disturbances, hot flushes | |
| Molindone | 5.15 | 1.58 |  | Atypical antipsychotic.  D_2_ and 5HT_7_ antagonist | I | Psychotic disorders | |
| Moperone | 7.26 | 2.81 |  | Neuroleptic typical antipsychotic.  D_2_, 5HT_2A_, H_1_ and SIGMAR1 antagonist | I | Schizophrenia, paranoid disorders | |
| Mosapride | 5.37 | 1.64 |  | Gastroprokinetic.  5HT_3_ inactivator and 5HT4 agonist | I | Chronic constipation, irritable bowel syndrome, epigastric pain | |
| Nafadotride | 7.27 | 3.01 |  | Atypical antipsychotic.  D_3_, D_4_ and weak D_2_ antagonist and 5HT_1A_ agonist | I | Schizophrenia, ADHD, catalepsy | |
| Nemonapride | 5.25 | 1.59 |  | Atypical antipsychotic.  D_3_, D_4_ and weak D_2_ antagonist | I | Schizophrenia | |
| Nonaperone | 7.09 | 2.45 |  | Neuroleptic, typical antipsychotic.  Dopamine antagonist | I | Psychotic disorders | |
| Nortriptyline | 5.13 | 1.37 |  | Tricyclic antidepressant.  D_2_, 5HT_1A_, 5HT_2A_, 5HT_2C_, α_1A_, α_1B_, α_1D_, α_2_, β, H_1_ and muscarinic antagonist, SERT and NET inhibitor, K_ir_4.1 blocker | II | Depressive disorders and infantile nocturnal enuresis | |
| (*R*)-Nuciferine | 5.72 | 1.82 |  | 5HT_7_ inverse agonist, 5HT_2A_, 5HT_2B_ and 5HT_2C_ antagonist, partial D_2_, D_5_ and 5HT_6_ agonist, 5HT_1A_ and D_4_ agonist and Ca_v_1 blocker [17] | II | Investigated for the treatment of psychotic disorders, bronchospastic respiratory diseases and in colorectal adenocarcinoma | |
| Ocaperidone | 6.45 | 2.43 |  | Atypical antipsychotic.  5HT_1B_ and 5HT_1D_ inverse agonist, D_2_ antagonist and 5HT_1A_ full agonist | I | Schizophrenia | |
| Olanzapine | 5.27 | 1.72 |  | Atypical antipsychotic.  5HT_2C_ and 5HT_6_ inverse agonist, D1, D_2_, D_3_, D_4_, D_5_, 5HT_2A_, 5HT_7_, α_1A_, α_1B_, α_2A_, α_2B_, α_2C_, H_1_, H_4_, M_1_, M_2_, M_3_, M_4_, M_5_ and β antagonist, benzodiazepine site-GABA_A_ and 5HT_3_ inactivator, and 5HT_1A_, 5HT_1B_, 5HT_1D_, 5HT_1E_ and 5HT_1F_ full agonist | I | Schizoprenia, bipolar disorders, psychomotor agitation, recurrent depressive disorder, delirium, Tourette’s syndrome, posttraumatic stress disorder and acute manic episode | |
| OSU-6162 | 6.19 | 1.77 |  | Partial D_2_ and 5HT_2A_ agonist | II | Investigated for the treatment of Parkinson’s disease, Huntington’s disease and schizophrenia | |
| Paliperidone | 5.89 | 1.78 |  | Atypical antipsychotic, metabolite of risperidone.  D_1_, D_2_, D_3_, D_4_, 5HT_1A_, 5HT_1D_, 5HT_2A_, 5HT_2C_, α_1A_, α_1B_, α_2A_, α_2B_ and H_1_ antagonist, and α_2C_ agonist | I | Schizophrenia and schizoaffective disorder | |
| Pardoprunox | 4.44 | 0.95 |  | Partial D_2_ and D_3_ agonist and 5HT_1A_ agonist | I | Investigated for the treatment of Parkinson's disease. | |
| PD-128,907 | 4.76 | 1.23 |  | Selective D_2_ and D_3_ full agonist [18] | I | Research |  |
| PD-168,077 | 6.28 | 2.16 |  | Partial D_4_ agonist | II | Research |  |
| Pentiapine | 5.61 | 1.68 |  | Dopamine antagonist | I | Research |  |
| Pergolide | 4.37 | 1.07 |  | Antiparkinsonian.  Partial D_3_, D_4_, 5HT_1A_, 5HT_1D_, 5HT_2C_, α_2A_, α_2B_ and α_2C_ agonist, and full D_1_, D_2_, D_5_, 5HT_1B_, 5HT_2A_, 5HT_2B_, 5HT_6_, 5HT_7_, α_1A_, α_1B_ and α_1D_ agonist | I | Parkinson’s disease, Cushing disease |  |
| Perospirone | 5.70 | 1.81 |  | Atypical antipsychotic.  5HT_2A_ and H_1_ inverse agonist, and D_2_, D_4_ and α_1_ antagonist | I | Schizophrenia and bipolar disorders |  |
| Perphenazine | 4.65 | 1.29 |  | Typical antipsychotic.  5HT_6_ and 5HT_7_ inverse agonist, D_1_, D_2_, 5HT_2A_, 5HT_2C_, M_1_, H_1_, α_1_ and α_2A_ antagonist, CaM blocker and binder of AR | II | Schizophrenia, acute mania, anxiety, depressive disorder, nausea and emesis |  |
| PF-216061 | 4.82 | 1.12 |  | Selective D_3_ agonist [19] | I | Research |  |
| PF-592379 | 5.04 | 1.35 |  | Selective D_3_ agonist | I | Research |  |
| Pimavanserin | 4.86 | 1.23 |  | Atypical antipsychotic.  5HT_2A_ inverse agonist | II | Schizophrenia |  |
| Pimethixene | 5.36 | 1.65 |  | Neuroleptic.  5HT_2A_, 5HT_2B_, H_1_ and M_1_ antagonist | I | Allergic conditions |  |
| Pimozide | 4.41 | 0.98 |  | Atypical antipsychotic.  D_2_, D_3_, 5HT_1A_, 5HT_2A_, 5HT_6_ and 5HT_7_ antagonist, and CaM blocker | II | Schizophrenia and Tourette’s syndrome |  |
| Pipamperone | 6.83 | 2.62 |  | Typical antipsychotic.  D_4_, D_1_, D_2_, 5HT_2A_, 5HT_2B_, 5HT_2C_, α_1_ and α_2A_ antagonist | I | Schizophrenia and depressive disorder |  |
| Pipothiazine | 5.65 | 2.07 |  | Neuroleptic, typical antipsychotic.  D_1_, D_2_, 5HT_1A_ and 5HT_2A_ antagonist, and CaM blocker | I | Schizophrenia |  |
| Piribedil | 5.61 | 1.77 |  | Partial D_2_, D_3_ and 5HT_1A_ agonist, and D_4_, 5HT_2B_, α_1A_, α_2A_ and α_2C_ antagonist | II | Parkinson’s disease and obliterant arteriopathy |  |
| Pramipexole | 3.97 | 0.77 |  | Antiparkinsonian.  D_2_, D_3_, D_4_, 5HT_1A_ and α_2A_ agonist, and carbonic anhydrase I inhibitor | I | Parkinson’s disease, restless legs syndrome |  |
| Prideperone | 6.33 | 2.03 |  | Antipsychotic | I | Psychotic disorders |  |
| Primaperone | 7.10 | 2.46 |  | Typical antipsychotic.  Serotonin antagonist | I | Psychotic disorders |  |
| Prochlorperazine | 4.63 | 1.35 |  | Typical antipsychotic.  D_1_, D_2_, D_3_, D_4_, 5HT_1A_ and 5HT_2A_ antagonist, and P2X_7_ inactivator | II | Schizophrenia, nausea and emesis, anxiety, migraine |  |
| Promethazine | 4.47 | 1.14 |  | Neuroleptic, typical antipsychotic.  D_2_, 5HT_2A_, α_1A_, H_1_, H_2_, M_1_, M_2_, M_3_, M_4_, M_5_ and P2Y antagonist | II | Nausea and emesis, motion sickness, severe benign paroxysmal positional vertigo |  |
| Propiomazine | 6.88 | 3.03 |  | Neuroleptic, typical antipsychotic.  D_2_, 5HT_2A_, 5HT_2C_, α_1A_, H_1_ and muscarinic antagonist | I | Insomnia, anxiety, restlessness |  |
| Propyperone | 7.37 | 3.33 |  | Neuroleptic and mammotropic agent | I | Research |  |
| (*R*)-Pukateine | 5.52 | 1.76 |  | D_1_ and D_2_ agonist | II | Research for the treatment of the Parkinson’s disease |  |
| Quetiapine | 5.72 | 1.88 |  | Atypical antipsychotic, thymomodulator, sedative antidepressant.  D_1_, D_2_, 5HT_6_, 5HT_2A_, H_1_, M_1_, M_3_, α_1_, α_2A_, α_2B_ and α_2C_, antagonist, partial 5HT_1A_ agonist, and 5HT_1D_ and 5HT_1F_ full agonist | I | Schizophrenia, bipolar disorders, obsessive-compulsive disorder, depressive disorders |  |
| Quinagolide | 4.32 | 0.88 |  | Antiparkinsonian. D_1_ and D_2_ agonist | I | Parkinson’s disease, hyperprolactinemia, galactorrhea |  |
| Quinelorane | 5.58 | 1.66 |  | D_3_ and D_2_ agonist | II | Investigated for the treatment of Parkinson’s disease, depressive disorders, anxiety, male sexual behavior promoter and in hypertension |  |
| Quinpirole | 3.87 | 0.53 |  | Selective D_2_ and D_3_ agonist, and also 5HT_1A_, 5HT_2A_, 5HT_2B_ and 5HT_2C_ full agonist | I | Research |  |
| Raclopride | 6.66 | 2.40 |  | D_2_, D_3_, D_4_ and 5HT_1A_ antagonist | I | Investigated for the treatment of Parkinson’s disease, Huntington’s disease, Alzheimer’s disease and depressive disorders |  |
| RDS-127 | 4.38 | 0.92 |  | D_2_ agonist | I | Research |  |
| Remoxipride | 5.33 | 1.46 |  | Antipsychotic.  D_2_, D_3_, D_4_ and SIGMAR1 antagonist | II | Schizophrenia |  |
| Renzapride | 5.04 | 1.38 |  | Gastroprokinetic.  5HT_4_ agonist and 5HT_3_ inactivator | II | Research for the treatment of irritable bowel syndrome, chronic constipation and colon pain/disconfort |  |
| Rilapine | 7.06 | 3.02 |  | 5HT_6_ antagonist | I | Research for psychotic disorders |  |
| Risperidone | 5.51 | 1.54 |  | Atypical antipsychotic.  5HT_2A_, 5HT_2C_, 5HT_6_ and 5HT_7_ inverse agonist, D_1_, D_2_, D_3_, D_4_, 5HT_1A_, 5HT_1D_, 5HT_2A_, 5HT_2C_, α_1A_, α_1B_, α_2A_, α_2B_, α_2C_ and H_1_ antagonist | I | Schizophrenia, bipolar disorders, depressive disorders, anorexia nervosa, borderline type emotionally unstable personality disorder, autistic spectrum disorders, acute mania |  |
| Ro10-5824 | 5.49 | 1.61 |  | Partial D_4_ agonist | II | Research for nootropic effects in animals |  |
| Ropinirole | 4.68 | 1.09 |  | Antiparkinsonian.  D_2_, D_3_ and D_4_ agonist, and α antagonist | I | Parkinson's disease and restless legs syndrome, sexual dysfunction |  |
| Rotigotine | 4.04 | 0.71 |  | Antiparkinsonian. D_1_, D_2_, D_3_, D_4_, D_5_ and 5HT_1A_ and agonist, and α_2B_ antagonist | I | Parkinson's disease and restless legs syndrome, with antidepressant effects |  |
| Roxindole | 5.09 | 1.60 |  | Psycholeptic, thymomodulator, sedative antidepressant.  Partial D_2_, D_3_, D_4_, 5HT_1A_, 5HT_1B_ and 5HT_1D_ agonist, and 5HT2A, 5HT2C, α_1A_, α_2A_ and α_2C_ antagonist | I | Depressive disorders with and without psychotic symptoms, Parkinson’s disease |  |
| Roxoperone | 7.09 | 2.45 |  | Antipsychotic. | I | Research |  |
| S-142907 | 4.44 | 1.05 |  | Dopamine antagonist | II | Research |  |
| Sarizotan | 5.89 | 1.94 |  | Partial D_2_ agonist and 5HT_1A_ full agonist | I | Investigated for the treatment of Parkinson’s disease and Rett Syndrome |  |
| SCH-23390 | 4.96 | 1.23 |  | Selective D_1_ and D_5_ antagonist | II | Research in Parkinson’s disease, amphetamine abuse and |  |
| Seridopidine | 6.33 | 1.88 |  | Dopaminergic ligand | I | Research in Parkinson’s disease and Tourette’s syndrome |  |
| Sertindole | 4.90 | 1.39 |  | Atypical antipsychotic, thymomodulator.  5HT_2C_ inverse agonist, D_2_, D_3_, D_4_, 5HT_1A_, 5HT_1B_, 5HT_1D_, 5HT_1F_, 5HT_2A_, 5HT_6_ and H_1_ antagonist | II | Schizophrenia |  |
| Setoperone | 6.98 | 2.69 |  | Antipsychotic.  5HT_2A_ antagonist | I | Schizophrenia, depressive disorders |  |
| SKF-38393 | 4.58 | 1.10 |  | Partial D_1_ and D_5_ agonist | I | Research |  |
| SKF-77434 | 4.38 | 0.97 |  | Partial D_1_ agonist | I | Research |  |
| SKF-81297 | 4.69 | 1.12 |  | Full D_1_ agonist | I | Research |  |
| SKF-82958 | 4.58 | 1.05 |  | D_1_ and ERα agonist | I | Research |  |
| SKF-83959 | 4.59 | 1.06 |  | Biased D_1_ agonist and SERT, NET and DAT inhibitor, and NaBC1 binder | I | Research |  |
| SKF-89145 | 4.67 | 1.14 |  | D_1_ agonist | I | Investigated for the treatment of parkinson’s disease and depressive disorders |  |
| Spiperone | 7.01 | 3.00 |  | Typical antipsychotic.  α_1B_ inverse agonist, D_2_, D_3_, D_4,_ D_5_, 5HT_1A_, 5HT_1B_, 5HT_1D_, 5HT_2A_, 5HT_2B_, 5HT_2C_, 5HT_6_, 5HT_7_, α_1A_ and α_1D_ antagonist, and CaCC activator | I | Schizophrenia, investigated for the treatment of cystic fibrosis [20] |  |
| Spiroxatrine | 4.21 | 0.92 |  | D_2_, 5HT_2B_, α_1_, and α_2_ antagonist, and 5HT_1A_ and ORL1 agonist | II | Research |  |
| Stepholidine | 4.37 | 0.97 |  | D_1_, D_2_, D_3_, D_4_ and D_5_ antagonist | I | Research antipsychotic |  |
| Sulpiride | 6.40 | 2.05 |  | Antipsychotic and antidepressant. D_2_ and D_3_ antagonist, and carbonic anhydrase 2 and 3 inhibitor. Also DNMT1 downregulator. | I | Schizophrenia and depressive disorders |  |
| Sumanirole | 4.50 | 1.01 |  | Antiparkinsonian.  Selective D_2_ receptor full agonist | I | Parkinson’s disease and restless legs syndrome |  |
| Talipexole | 4.04 | 0.80 |  | Antiparkinsonian.  D_2_, D_3_, D_4_, α_2A_, α_2B_ and α_2C_ agonist | I | Parkinson’s disease |  |
| Teflutixol | 5.39 | 1.59 |  | Dopamine antagonist | I | Research |  |
| Tenilapine | 7.57 | 3.25 |  | 5HT_2C_ inverse agonist and D_4_ antagonist | I | Research |  |
| Tetrabenazine | 5.52 | 1.65 |  | Antihyperkinetic.  D_2_ antagonist and SVAT inhibitor | I | Huntington’s disease |  |
| Thiethylperazine | 4.20 | 1.05 |  | Typical antipsychotic and antiemetic.  D_1_, D_2_, D_4_ antagonist and ABCC1 activator [21] | II | Nausea and emesis |  |
| Thioridazine | 4.20 | 1.03 |  | Neuroleptic, typical antipsychotic.  5HT_6_ and 5HT_7_ inverse agonist, and D_1_, D_2_, D_5_, 5HT_1A_, 5HT_2A_, 5HT_2C_, α_1A_, α_2A_ and H_1_ antagonist | II | Schizophrenia |  |
| Thiothixene | 6.10 | 2.18 |  | Typical antipsychotic.  D_1_, D_2_, 5HT_2A_ and H_1_ antagonist | I | Schizophrenia |  |
| Tiapride | 6.22 | 1.90 |  | Antipsychotic, antihyperkinetic.  D_2_, D_3_, D_4_, α_1_, α_2_ and serotonin antagonist | I | Huntington’s disease, Tourette syndrome and dyskinesias |  |
| Timiperone | 7.12 | 3.10 |  | Typical antipsychotic.  D_2_ antagonist | I | Schizophrenia |  |
| Tiospirone | 5.70 | 1.81 |  | Atypical antipsychotic.  5HT_2C_ inverse agonist, 5HT_6_ and 5HT_7_ antagonist and partial 5HT_1A_ agonist | I | Schizophrenia |  |
| Trepipam | 4.61 | 0.93 |  | Psycholeptic.  D_1_ antagonist | I | Schizophrenia, aggressive behavior |  |
| Trifluoperazine | 5.12 | 1.66 |  | Typical antipsychotic.  D_1_, D_2_, D_3_, D_4_, 5HT_2A_, 5HT_2C_, α_1A_, H_1_ and calcyon antagonist, and CaM blocker | I | Schizophrenia, anxiety disorders |  |
| Trifluperidol | 7.10 | 2.46 |  | Typical antipsychotic.  D_2_, D_3_, D_4_, α_1_, α_2A_, 5HT_2B_ and SIGMAR1 antagonist, NMDAR inactivator and 5HT_2A_ agonist | I | Schizophrenia, bipolar disorders, acute mania |  |
| UH-232 | 5.88 | 1.91 |  | D_2_ and D_3_ binder | I | Research |  |
| Veralipride | 6.73 | 2.28 |  | Antipsychotic, vasomodulator.  D_2_ and D_3_ antagonist | I | Hot flashes |  |
| Vilazodone | 6.41 | 2.46 |  | Atypical, sedative antidepressant.  Partial 5HT_1A_ agonist, D_2_, D_3_ and 5HT_4_ full agonist, and DAT and SERT inhibitor | II | Major depressive disorder |  |
| Yohimbine | 4.54 | 1.14 |  | D_2_, D_3_, 5HT_1B_, 5HT_1D_, 5HT_2A_, 5HT_2B_, 5HT_2C_, α_2A_, α_2B_ and α_2C_ antagonist, and weak 5TH_1A_ agonist | II | Research |  |
| Zacopride | 5.14 | 1.44 |  | Gastroprokinetic.  5HT_4_ agonist and 5HT_3_ inactivator | II | Nausea and emesis, investigated for the treatment of sleep apnea |  |
| Zelandopam | 4.41 | 0.97 |  | D_1_ agonist | I | Congestive heart failure, acute renal failure |  |
| Zetidoline | 4.71 | 1.09 |  | Neuroleptic.  D_2_ and α_1_ antagonist | II | Psychotic disorders |  |
| Zicronapine | 5.40 | 1.51 |  | Antipsychotic. | I | Investigated for the treatment of schizophrenia |  |
| Ziprasidone | 5.70 | 1.81 |  | Atypical antipsychotic.  5HT_2C_ and 5HT_7_ inverse agonist, D_1_, D_2_, D_3_, D_4_, D_5_, 5HT_2A_, 5HT_6_, , α_1A_, α_1B_, α_2A_, α_2B_, α_2C_, H_1_, M_1_, M_2_, M_3_, M_4_, M_5_ antagonist, and 5HT_1A_, 5HT_1B_, 5HT_1D_ and 5HT_1E_ agonist | I | Schizophrenia, bipolar disorders, acute mania |  |
| Zoloperone | 5.11 | 1.44 |  | Antipsychotic | II | Research |  |
| Zuclopenthixol | 5.81 | 2.00 |  | Typical antipsychotic.  D_1_, D_2_, D_5_, 5HT_2A_, α_1A_, α_2A_ and H_1_ antagonist | I | Schizophrenia and bipolar disorders |  |

**References**

[1] NCATS, NCATS Creates Drug Development Data Portal, Natl. Cent. Adv. (2020).

[2] S.D. Harding, J.L. Sharma, E. Faccenda, C. Southan, A.J. Pawson, S. Ireland, A.J.G. Gray, L. Bruce, S.P.H. Alexander, S. Anderton, C. Bryant, A.P. Davenport, D. C., D. Fabbro, F. Levi-Schaffer, M. Spedding, J.A. Davies, NC-IUPHAR, The IUPHAR/BPS Guide to PHARMACOLOGY in 2018: updates and expansion to encompass the new guide to IMMUNOPHARMACOLOGY, Nucleic Acids Res. 46 (2018) D1091–D1106.

[3] J.M. Beaulieu, E. Borrelli, A. Carlsson, M.G. Caron, O. Civelli, S. Espinoza, G. Fisone, R.R. Gainetdinov, D.K. Grandy, J.W. Kebabian, S.Z. Langer, M.C. Missale, K.A. Neve, B. Scatton, J.C. Schwartz, G. Sedvall, P. Seeman, D.R. Sibley, P. Sokoloff, P.F. Spano, H.H.M. Van Tol, Dopamine receptors (version 2019.4) in the IUPHAR/BPS Guide to Pharmacology Database, IUPHAR/BPS Guid. to Pharmacol. CITE, 2019(4). (2019).

[4] D.S. Wishart, Y.D. Feunang, A.C. Guo, E.J. Lo, A. Marcu, J.R. Grant, T. Sajed, D. Johnson, C. Li, Z. Sayeeda, N. Assempour, I. Iynkkaran, Y. Liu, A. Maciejewski, N. Gale, A. Wilson, L. Chin, R. Cummings, D. Le, A. Pon, C. Knox, M. Wilson, DrugBank 5.0: a major update to the DrugBank database for 2018, Nucleic Acids Res. 46 (2018) D1074–D1082.

[5] A. Karlsson, L. Björk, L. Pettersson, N.E. Andén, U. Hacksell, (R)- and (S)-5-hydroxy-2-(dipropylamino)tetralin (5-OH-DPAT): assessment of optical purities and dopaminergic activities, Chirality. 2 (1990) 90–95.

[6] X. Lamas, S.S. Negus, M.A. Nader, N.K. Mello, Effects of the putative dopamine D3 receptor agonist 7-OH-DPAT in rhesus monkeys trained to discriminate cocaine from saline, Psychopharmacology (Berl). 124 (1996) 306–314.

[7] J.B. Acri, S.R. Carter, K. Alling, B. Geter-Douglass, D. Dijkstra, H. Wikström, J.L. Katz, J.M. Witkin, Assessment of cocaine-like discriminative stimulus effects of dopamine D3 receptor ligands, Eur. J. Pharmacol. 281 (1995) R7-9.

[8] M.P. Kung, S. Chumpradit, D. Frederik, S. Garner, K.D. Burris, P.B. Molinoff, H.F. Kung, Characterization of binding sites for [125I]R(+)trans7-OH-PIPAT in rat brain, Naunyn-Schmiedebergs Arch. Pharmacol. 350 (1994) 611–7.

[9] M.E. Goldman, J.W. Kebabian, Aporphine enantiomers. Interactions with D-1 and D-2 dopamine receptors, Mol. Pharmacol. 25 (1984) 18–23.

[10] J.M. Schaus, R.D. Titus, M.M. Foreman, N.R. Mason, L.L. Truex, Aporphines as Antagonists of Dopamine D-1 Receptors, J. Med. Chem. 33 (1990) 600–607.

[11] A. Zhang, R. Zhang, A.R. Branfman, R.J. Baldessarini, J.L. Neumeyer, Advances in Development of Dopaminergic Aporphinoids, J. Med. Chem. 50 (2007) 171–181.

[12] M.H. Hedberg, T. Linnaen, J.M. Jansen, G. Nordvall, S. Hjorth, L. Unelius, A.M.J. Johansson, 11-Substituted (R)-Aporphines: Synthesis, Pharmacology, and Modeling of D2A and 5HT1A Receptor Interactions, J. Med. Chem. 39 (1996) 3503–3513.

[13] C. Yi, P. Ezan, P. Fernández, J. Schmitt, J.C. Sáez, C. Giaume, A. Koulakoff, Inhibition of glial hemmichannels by boldine treatment reduces neuronal suffering in a murine model of Alzheimer’s disease, Glia. (2017) 1–19.

[14] H.A. Lashuel, D.M. Hartley, D. Balakhaneh, A. Aggarwal, S. Teichberg, D.J. Callaway, New Class of inhibitors of amyloid-beta fibril formation. Implications for the mechanism of pathogenesis in Alzheimer’s disease, J. Biol. Chem. 277 (2002) 42881–42890.

[15] A. Fink-Jensen, E.B. Nielsen, L. Hansen, M.A. Scheideler, Behavioral and neurochemical effects of the preferential dopamine D3 receptor agonist cis-8-OH-PBZI, Eur. J. Pharmacol. 342 (1998) 153–61.

[16] R.K. Sunahara, H.-C. Guan, B.F. O’Dowd, P. Seeman, L.G. Laurier, G. Ng, S.R. George, J. Torchia, H.H.M. van Tol, H.B. Niznik, Cloning of the gene for a human D5 receptor with higher affinity for dopamine than D1, Lett. to Nat. 350 (1991) 614–619.

[17] M.S. Farrell, J.D. McCorvy, X.-P. Huang, D.J. Urban, K.L. White, P.M. Guigere, K. Doak, A, A.I. Bernstein, K.A. Stout, S.M. Park, R.M. Rodriguiz, B.W. Gray, W.S. Hyatt, A.P. Norwood, K.A. Webster, B.M. Gannon, G.W. Miller, J.H. Porter, B.K. Shoichet, W.E. Fantegrossi, W.C. Wetsel, B.L. Roth, In vitro and in vivo characterization of the alkaloid nuciferine, PLoS One. 11 (2016) e0150602.

[18] H.A. DeWald, T.G. Heffner, J.C. Jaen, D.M. Lustgarten, A.T. McPhail, L.T. Meltzer, T.A. Pugsley, L.D. Wise, Synthesis and dopamine agonist properties of (+)-trans-3,4,4a,10b-tetrahydro-4-propyl-2H,5H-[1]benzopyrano [4,3-b]-1,4-oxazin-9-ol and its enantiomers, J. Med. Chem. 33 (1990) 445–450.

[19] J. Blagg, C.M.N. Allerton, D.V.J. Batchelor, A.D. Baxter, D.J. Burring, C.L. Carr, A.S. Cook, C.L. Nichols, J. Phipps, V.G. Sanderson, H. Verrier, S. Wong, Design and synthesis of a functionally selective D3 agonist and its in vivo delivery via the intranasal route, Bioorganic Med. Chem. Lett. 17 (2007) 6691–6.

[20] L. Liang, K. MacDonald, E.M. Schwiebert, P.L. Zeitlin, W.B. Guggino, Spiperone, identified through compounds screening, activates calcium dependent chloride secretion in the airway, Am. J. Physiol. Cell Physiol. 296 (2009) C131-41.

[21] M. Krohn, C. Lange, J. Hofrichter, K. Scheffler, J. Stenzel, J. Steffen, T. Schumacher, T. Brüning, A.-S. Plath, F. Alfen, A. Schmidt, F. Winter, K. Rateitschak, A. Wree, J. Gsponer, L.C. Walker, J. Pahnke, Cerebral amyloid-β proteostasis is regulated by the membrane transport protein ABCC1 in mice, J. Clin. Invest. 121 (2011) 3924–3931.

## References of the tables

## 1. Karlsson A, Björk L, Pettersson L, Andén NE, Hacksell U. (R)- and (S)-5-hydroxy-2-(dipropylamino)tetralin (5-OH-DPAT): assessment of optical purities and dopaminergic activities. Chirality. 1990;2(2):90–5.

## 2. Beaulieu JM, Borrelli E, Carlsson A, Caron MG, Civelli O, Espinoza S, et al. Dopamine receptors (version 2019.4) in the IUPHAR/BPS Guide to Pharmacology Database. IUPHAR/BPS Guide to Pharmacology CITE, 2019(4). 2019.

## 3. Neumeyer JL, Baindur N, Niznik HB, Guan HC, Seeman P. (.+-.)-3-Allyl-6-bromo-7,8-dihydroxy-1-phenyl-2,3,4,5-tetrahydro-1H-3-benzazepine, a new high affinity D1 dopamine receptor ligand: synthesis and structure-activity relationship. J Med Chem. 1991;34(12):3366–3371.

## 4. Lamas X, Negus SS, Nader MA, Mello NK. Effects of the putative dopamine D3 receptor agonist 7-OH-DAT in rhesus monkeys trained to discriminate cocaine from saline. Psychopharmacology (Berl). 1996;124(4):306–14.

## 5. Acri JB, Carter SR, Alling K, Geter-Douglass B, Dijkstra D, Wikström H, et al. Assessment of cocaine-like discriminative stimulus effects of dopamine D3 receptor ligands. Eur J Pharmacol. 1995;281(2):R7-9.

## 6. Kung MP, Chumpradit S, Frederik D, Garner S, Burris KD, Molinoff PB, et al. Characterization of binding sites for [125I]R(+)trans7-OH-PIPAT in rat brain. Naunyn-Schmiedebergs Arch Pharmacol. 1994;350(6):611–7.

## 7. Moreland RB, Patel M, Hsieh GC, Wetter JM, Marsh K, Brioni JD. A-412997 is a selective dopamine D4 receptor agonist in rats. Pharmacol Biochem Behav. 2005;82(1):140–7.

## 8. NCATS. NCATS Creates Drug Development Data Portal. National Center for Advancing. 2020.

## 9. Kebabian JW, DeNinno MP, Schoenleber R, MacKenzie R, Britton DR, Asin KE. A-77636: A potent agonist specific for the dopamine D1 receptor. Neurochem Int. 1992;20(SUPPL.):157–60.

## 10. Michaelides MR, Hong Y, DiDomenico S, Asin KE, Britton DR, Lin CW, et al. (5aR,11bS)-4,5,5a,6,7,11b-hexahydro-2-propyl-3-thia-5-azacyclopent-1- ena[c]-phenanthrene-9,10-diol (A-86929): a potent and selective dopamine D1 agonist that maintains behavioral efficacy following repeated administration and characterization of its diac. J Med Chem. 1995;38(18):3445–7.

## 11. Seneca M, Finnema SJ, Farde L, Gulyás B, Wikström H V., Halldin C, et al. Effect of Amphetamine on Dopamine D2 Receptor Binding in Nonhuman Primate Brain: A Comparison of the Agonist Radioligand [11C]MNPA and Antagonist [11C]Raclopride. Synapse. 2006;59(5):260–9.

## 12. Wishart DS, Feunang YD, Guo AC, Lo EJ, Marcu A, Grant JR, et al. DrugBank 5.0: a major update to the DrugBank database for 2018. Nucleic Acids Res. 2018;46(D1):D1074–82.

## 13. DAILYMED. DailyMed is the official provider of FDA label information. U. S. NATIONAL LIBRARY OF MEDICINE. 2020.

## 14. Watanabe H, Somei M, Segihara S, Nakagawa K, Yamada F. Dopamine Receptor Stimulating Effects of Chanoclavine Analogues, Tricyclic Ergot Alkaloids, in the Brain. Jpn J Pharmacol. 1987;45(4):501–6.

## 15. Fink-Jensen A, Nielsen EB, Hansen L, Scheideler MA. Behavioral and neurochemical effects of the preferential dopamine D3 receptor agonist cis-8-OH-PBZI. Eur J Pharmacol. 1998;342(2–3):153–61.

## 16. Burns MJ, Linden CH. Antipsychotic Poisoning. In: Irwin RS, Rippe JM, editors. Irwin and Rippe’s Intensive Care Medicine. Sixth. Lippincott Williams & Wilkins; 2008. p. 1525–6.

## 17. Gulwadi AG, Korpinen CD, Mailman RB, Nichols DE, Sit SY, Taber MT. Dinapsoline: Characterization of a D1 Dopamine Receptor Agonist in a Rat Model of Parkinson’s Disease. J Pharmacol Exp Ther. 2001;296(2):338–44.

## 18. Franck J. Pharmacotherapy for Alcohol Withdrawal Syndrome. In: Berglund M, Thelander S, Jonsson E, editors. Treating Alcohol and Drug Abuse: An Evidence Based Review. WILEY-VCH,; 2003.

## 19. Harding SD, Sharma JL, Faccenda E, Southan C, Pawson AJ, Ireland S, et al. The IUPHAR/BPS Guide to PHARMACOLOGY in 2018: updates and expansion to encompass the new guide to IMMUNOPHARMACOLOGY. Nucleic Acids Res. 2018;46(D1):D1091–106.

## 20. Jolly E, Clayton A, Thorp J, Lewis-D’Agostino D, Wunderlich G, Lesko L. T09-P-05 Design of Phase III pivotal trials of flibanserin in female Hypoactive Sexual Desire Disorder (HSDD). Sexologies. 2008;17(1):S133–4.

## 21. Cascio G, Manghisi E, Fregnan G. 5-Piperazinylalkyl-2(3H)-oxazolones With Neuroleptic Activity. J Med Chem. 1989;32(10):2241–7.

## 22. ClinicalTrials.gov. ClinicalTrials.gov is a database of privately and publicly funded clinical studies conducted around the world. U. S. NATIONAL LIBRARY OF MEDICINE. 2020.

## 23. DeWald HA, Heffner TG, Jaen JC, Lustgarten DM, McPhail AT, Meltzer LT, et al. Synthesis and dopamine agonist properties of (+)-trans-3,4,4a,10b-tetrahydro-4-propyl-2H,5H-[1]benzopyrano [4,3-b]-1,4-oxazin-9-ol and its enantiomers. J Med Chem. 1990;33(1):445–50.

## 24. Blagg J, Allerton CMN, Batchelor DVJ, Baxter AD, Burring DJ, Carr CL, et al. Design and synthesis of a functionally selective D3 agonist and its in vivo delivery via the intranasal route. Bioorganic Med Chem Lett. 2007;17(24):6691–6.

## 25. Collins GT, Butler P, Wayman C, Ratcliffe S, Gupta P, Oberhofer G, et al. Lack of Abuse Potential in a Highly Selective Dopamine D3 Agonist, PF-592,379, in Drug Self-Administration and Drug Discrimination in Rats. Behav Pharmacol. 2012;23(3):280–91.

## 26. Kvernmo T, Härtter S, Burger E. A review of the receptor-binding and pharmacokinetic properties of dopamine agonists. Clin Ther. 2006;28(8):1065–78.

## 27. Chan-Tack KM. Neuroleptic Malignant Syndrome Due to Promethazine. South Med J. 1999;92(10):1017–8.

## 28. Eilam D, Szechtman H. Biphasic effect of D-2 agonist quinpirole on locomotion and movements. Eur J Pharmacol. 1989;161(2–3):151–7.

## 29. Arnerić SP, Long JP, Williams M, Goodale DB, Mott J, Lakoski JM, et al. RDS-127 (2-di-n-propylamino-4,7-dimethoxyindane): central effects of a new dopamine receptor agonist. J Pharmacol Exp Ther. 1983;224(1):161–70.

## 30. Eden RJ, Costall B, Domeney AM, Gerrard PA, Harvey CA, Kelly ME, et al. Preclinical pharmacology of ropinirole (SK&F 101468-A) a novel dopamine D2 agonist. Pharmacol Biochem Behav. 1991;38(1):147–54.

## 31. Chen JJ, Swope DM, Dashtipour K, Lyons KE. Transdermal rotigotine: A clinically innovative dopamine-receptor agonist for the management of Parkinson’s disease. Pharmacotherapy. 2009;29(12):1452–67.

## 32. Millan MJ, Peglion JL, Viat J, Rivet JM, Brocco M, Gobert A, et al. Functional Correlates of Dopamine D3 Receptor Activation in the Rat in Vivo and Their Modulation by the Selective Antagonist, (+)-S 14297: 1. Activation of Postsynaptic D3 Receptors Mediates Hypothermia, Whereas Blockade of D2 Receptors Elicits Prolactin . J Pharmacol Exp Ther. 1995;275(2):885–98.

## 33. Derlet R, Albertson TE, Rice P. The Effect of SCH 23390 Against Toxic Doses of Cocaine, d-Amphetamine and Methamphetamine. Life Sci. 1990;47(9):821–827.

## 34. Dobois A, Savasta M, Curet O, Scatton B. Autoradiographic distribution of the D1 agonist [3H]SKF 38393, in the rat brain and spinal cord. Comparison with the distribution of D2 dopamine receptors. Neuroscience. 1986;19(1):125–37.

## 35. Pfeiffer FR, Wilson JW, Weinstock J, Kuo GY, Chambers PA, Holden KG, et al. Dopaminergic activity of substituted 6-chloro-1-phenyl-2,3,4,5-tetrahydro-1H-3-benzazepines. J Med Chem. 1982;25(4):352–358.

## 36. Platt DM, Rowlett JK, Spealman RD. Dissociation of cocaine-antagonist properties and motoric effects of the D1 receptor partial agonists SKF 83959 and SKF 77434. J Pharmacol Exp Ther. 2000;293(3):1017–26.

## 37. Weed MR, Vanover KE, Woolverton WL. Reinforcing effect of the D1 dopamine agonist SKF 81297 in rhesus monkeys. Psychopharmacology (Berl). 1993;113(1):51–2.

## 38. Self DW, Stein L. The D1 agonists SKF 82958 and SKF 77434 are self-administered by rats. Brain Res. 1992;582(2):349–52.

## 39. O’Boyle KM, Waddington JL. New substituted 1-phenyl-3-benzazepine analogues of SK&F 38393 and N-methyl-thienopyridine analogues of dihydroxynomifensine with selective affinity for the D-1 dopamine receptor in human post-mortem brain. Neuropharmacology. 1987;26(12):1807–10.

## 40. McCall RB, Lookingland KJ, Bédard PJ, Huff RM. Sumanirole, a highly dopamine D2-selective receptor agonist: in vitro and in vivo pharmacological characterization and efficacy in animal models of Parkinson’s disease. J Pharmacol Exp Ther. 2005;314(3):1248–56.

## 41. Krohn M, Lange C, Hofrichter J, Scheffler K, Stenzel J, Steffen J, et al. Cerebral amyloid-β proteostasis is regulated by the membrane transport protein ABCC1 in mice. J Clin Invest. 2011;121(10):3924–3931.

## 42. Borsini F, Nowakowska E, Samanin R. Effect of Repeated Treatment With Desipramine in the Behavioral “Despair” Test in Rats: Antagonism by “Atypical” but Not “Classical” Neuroleptics or Antiadrenergic Drugs. Life Sci. 1984;34(12):1171–6.

## 43. Goldman ME, Kebabian JW. Aporphine enantiomers. Interactions with D-1 and D-2 dopamine receptors. Mol Pharmacol. 1984;25(1):18–23.

## 44. Zhang A, Zhang R, Branfman AR, Baldessarini RJ, Neumeyer JL. Advances in Development of Dopaminergic Aporphinoids. J Med Chem. 2007;50(2):171–181.

## 45. Benber HC, Feldmann H. AHR 6134--a New Anxiolytic Drug With Unusual Clinical Properties. Prog Neuropsychopharmacol. 1978;2(1):117–22.

## 46. Carpenter S, Berk M, Rathbone J. Clotiapine for Acute Psychotic Illnesses. Cochrane Database Syst Rev. 2004;18(4):CD002304.

## 47. Dong E, Nelson M, Grayson DR, Costa E, Guidotti A. Clozapine and Sulpiride but Not Haloperidol or Olanzapine Activate Brain DNA Demethylation. Proc Natl Acad Sci United States Am. 2008;105(36):13614–9.

## 48. Li P, Zhang Q, Robichaud AJ, Lee T, Tomesch J, Yao W, et al. Discovery of tetracyclic quinoxaline derivative as a potent and orally active multifunctional drug candidate for the tratment of neuropsychiatric and neurological disorders. J Med Chem. 2014;57(6):2670–2682.

## 49. Pilla M, Perachon S, Sautel F, Garrido F, Mann A, Wermuth CG, et al. Selective inhibition of cocaine-seeking behaviour by a partial dopamine D3 agonist. Nature. 1999;400(6742):371–5.

## 50. Farrell MS, McCorvy JD, Huang X-P, Urban DJ, White KL, Guigere PM, et al. In vitro and in vivo characterization of the alkaloid nuciferine. PLoS One. 2016;11(3):e0150602.

## 51. Matulenko MA, Hakeem AA, Kolasa T, Nakane M, Terranova MA, Uchic ME, et al. Synthesis and functional activity of (2-aryl-1-piperazinyl)-N-(3-methylphenyl)acetamides: selective dopamine D4 receptor agonists. Bioorg Med Chem. 2004;12(13):3471–83.

## 52. van Craenenbroeck K, Gellynck E, Lintermans B, Leysen JE, van Tol HHM, Haegeman G, et al. Influence of the antipsychotic drug pipamperone on the expression of the dopamine D4 receptor. Life Sci. 2006;80(1):74–81.

## 53. Millan MJ, Cussac D, Milligan G, Carr C, Audinot V, Gobert A, et al. Antiparkinsonian agent piribedil displays antagonist properties at native, rat, and cloned, human alpha(2)-adrenoceptors: cellular and functional characterization. J Pharmacol Exp Ther. 2001;297(3):876–87.

## 54. Dajas-Bailador FA, Asencio M, Bonilla C, Scorza MC, Echeverry C, Reyes-Parada M, et al. Dopaminergic pharmacology and antioxidant properties of pukateine, a natural product lead for the design of agents increasing dopamine neurotransmission. Gen Pharmacol. 1999;32(3):373–9.

## 55. Powell SB, Paulus MP, Hartman DS, Godel T, Geyer MA. RO-10-5824 is a selective dopamine D4 receptor agonist that increases novel object exploration in C57 mice. Neuropharmacology. 2003;44(4):473–81.

## 56. Bartoszyk GD, Harting J, Minck KO. Roxindole: psychopharmacological profile of a dopamine D2 autoreceptor agonist. J Pharmacol Exp Ther. 1996;276(1):41–8.

## 57. Liang L, MacDonald K, Schwiebert EM, Zeitlin PL, Guggino WB. Spiperone, identified through compounds screening, activates calcium dependent chloride secretion in the airway. Am J Physiol Cell Physiol. 2009;296(1):C131-41.

## 58. Svendsen O. Long term effect of teflutixol on apomorphine-induced stereotypy and vomiting in dogs. Eur J Pharmacol. 1979;53(4):387–90.
